# Migraine Pain Location and Measures of Healthcare Use and Distress: An Observational Study

**DOI:** 10.1155/2018/6157982

**Published:** 2018-06-04

**Authors:** Elizabeth Loder, Emma Weizenbaum, Donald Giddon

**Affiliations:** ^1^Division of Headache, Department of Neurology, Brigham and Women's Faulkner Hospital, Harvard Medical School, Boston, MA, USA; ^2^John R. Graham Headache Center, Brigham and Women's Faulkner Hospital, Boston, MA, USA; ^3^Department of Psychology, Boston University, Boston, MA, USA; ^4^Harvard School of Dental Medicine, Department of Anesthesiology, Perioperative and Pain Medicine, Pain Management Center, Brigham and Women's Hospital, Boston, MA, USA

## Abstract

**Introduction:**

Lateralized pain is a core diagnostic feature of migraine. In previous research, left-sided spinal pain was more frequent and associated with greater emotional distress and healthcare use than right-sided pain. We hypothesized therefore that patients with left-sided head pain might experience higher levels of distress or healthcare use than those with right-sided or bilateral pain.

**Methods:**

Medical record information was extracted for 477 randomly selected patients with migraine seen in 2011 in a tertiary headache clinic. This included demographic data, pain location, handedness, comorbid psychiatric diagnoses, medical and emergency department visits, and use of selected headache medications.

**Results and Discussion:**

Two hundred twenty-eight of four hundred seventy-seven (47.8%) patients reported lateralized pain, of which 107 (47.9%) patients were right sided compared with 65 (28.5%) left-sided patients (*p*=0.001), while 56 (24.5%) reported unilateral pain with no side predominance. Contrary to expectations, with the exception of self-reported posttraumatic stress disorder, there were no statistically significant differences between left and right in measures of psychiatric distress, emergency department visits, or healthcare use.

**Conclusion:**

Although unilateral pain location can be helpful in making a migraine diagnosis, it does not appear to have additional clinical implications. Additionally, its absence does not rule out a diagnosis of migraine since more than half of migraineurs have bilateral head pain.

## 1. Introduction

A substantial body of research supports the view that trigeminal pain processing, like many other brain functions, is strongly lateralized to the right side. Functional imaging and other studies in a variety of pain disorders show greater activation of the right than left cerebral hemispheres [1–5]. Such observations are “consistent with the idea that there may be a right-lateralized attentional system to alert an organism to an infrequent, but behaviorally relevant, stimulus such as pain” [6]. Recent work suggests, however, that the cortical representation of trigeminal pain is more complex and inconsistent. Bilateral activation may result not only from physical stimulation but also from anticipation of pain [7].

The “right hemisphere hypothesis of emotion” proposes that cognitive processes are preferentially handled by the left hemisphere and emotional processes by the right hemisphere, although the results of recent research have been conflicting. An alternative hypothesis, referred to as the “valence asymmetry hypothesis,” holds that positive emotions are lateralized to the left hemisphere and negative emotions to the right. Yet another hypothesis, referred to as “region-specific functional lateralization,” suggests that emotional processing is frequently lateralized, but that the direction of lateralization depends upon the region of the brain [8, 9]. Since pain arising on the left side of the body is chiefly processed by the right side of the brain, it is plausible that left-sided pain may be more emotionally distressing and disabling than comparable pain arising on the right.

In prior research involving healthy subjects, pain thresholds for mechanical, thermal, or electrical stimulation were found to be lower on the left side of the body compared with the right [10–13]. Left-sided back pain, for example, was associated with higher levels of anxiety and depression compared to right-sided pain [14]. One study found that headaches and other pain occurred more frequently on the left side in patients with known somatization disorders [15]. Left-sided pain also has been associated with higher hypochondriasis scores on the Minnesota Multiphasic Personality Inventory and on the Sickness Impact Profile [16]. The majority of patients in the back pain study were men, and most of the previous studies examined pain of spinal nerve origin. It is thus unclear whether these findings would hold true in other types of lateralized pain, in women, or with pain that involves cranial rather than spinal nerves.

Unilateral pain location is a characteristic feature of migraine, but it is not present in all patients. To satisfy diagnostic criteria for migraine, patients must report two of the following four pain characteristics: unilateral location, moderate to severe intensity, throbbing quality, and aggravation by physical activity [17]. Previous studies suggest that roughly one half to two thirds of patients with migraine have unilateral pain, although this is confounded because unilateral location of pain is among the features used to make a migraine diagnosis.

Because migraine is a highly prevalent chronic disorder, it is an ideal condition in which to explore the association of pain laterality with distress, disability, and healthcare use. The association between pain lateralization and clinical outcomes has not previously been studied in headache. Additionally, the extent to which patients with lateralized head pain can be identified in routinely collected medical record data is unknown.

Based on these observations, we hypothesized that patients with head pain occurring exclusively or principally on the left side of the head would be having higher levels of distress, as measured by diagnoses of depression, anxiety, or other psychiatric comorbidities and would be more likely to use ED and other healthcare services. In the context of this paper, we use the word “distress” to mean “pain or suffering affecting the body, a bodily part, or the mind” [18]. These hypotheses were therefore evaluated in a large population of migraineurs in a tertiary headache clinic. The objectives were to examine the feasibility of extracting information on headache laterality from routinely collected electronic medical record data, describe the distribution of headache location and laterality within the study population, and then determine the association between lateralized head pain and selected measures of distress and healthcare use.

## 2. Materials and Methods

Following approval by the Institutional Review Board of Brigham and Women's Faulkner Hospital, the proprietary Partners Healthcare Physician On-Line Reporting (POLR) system database was used to obtain medical record numbers for a representative sample of patients listed as having received ICD-9 diagnostic codes for migraine, cluster headache, or hemicrania continua at the John R. Graham Headache Center of the Brigham and Women's Faulkner Hospital in Boston. The system captures physician-assigned diagnostic codes and other administrative information from all visits to Partners Healthcare facilities and can be queried to obtain information for specific practice locations, diagnoses, physicians, and dates. To obtain pilot data for a predetermined sample size calculation, medical record information was examined on 50 randomly selected patients with these diagnoses seen in 2009. For the final sample, the data used was from 500 randomly selected patients with these diagnoses seen in 2011.

Brigham and Women's Faulkner Hospital is part of the Partners Healthcare network, which provides care to approximately 50% of patients in the greater Boston metropolitan area. The headache clinic is a tertiary clinic providing care to patients referred from the Partners network. Some patients are referred from within the New England region or other parts of the United States. In 2011, there were 3619 patient visits to the Headache Center, consisting of 966 new patient visits and 2653 return visits. Clinic administrative staff enter demographic information and diagnostic codes into POLR for all patients seen in the headache clinic. Clinic physicians assign a diagnostic code or codes at every patient encounter, using the International Classification of Diseases, Ninth Revision (ICD-9).

For the pilot phase of the project, 50 patients were randomly selected by entering medical record numbers of all patients with diagnoses of interest seen in 2009 into a Microsoft Excel® spreadsheet. To generate a random sample of these patients, each record was assigned a random number, after which records were sorted by random number, and the first 50 were chosen for the pilot study. For the principal data collection phase of the study, this procedure was repeated to generate a random sample of 500 patients with diagnoses of interest seen in the clinic in 2011.

Medical records for these patients were identified in the Longitudinal Medical Record (LMR) system. LMR is a proprietary electronic medical record system developed by Partners Healthcare and used in Partners facilities. It captures information traditionally included in paper medical records, including demographic information and detailed, clinician-generated notes for medical encounters. The system records medications, allergies, medical problem lists, and other information and includes prescribing software that allows clinicians to electronically prescribe medications and other treatments.

### 2.1. Data Collection

A data collection form was created using Research Electronic Data Capture (REDCap) software. A single investigator (EW) abstracted information from each patient record using this data collection form. A subset of 50 records was reviewed by a second investigator (EL) to establish consensus about data extraction. For each patient record, the first headache consultation was identified. The diagnosis at this visit was verified as migraine, cluster headache, or hemicrania continua. Additional information was collected from the medical record and entered into the REDCap database. Information included basic demographic data (e.g., age, sex, highest level of education, marital, and employment status). Physician notes were obtained on (1) the location of headache pain, (2) handedness, (3) comorbid psychiatric diagnoses, (4) patient-initiated interactions with the healthcare system, (5) emergency department visits, and (6) use of selected headache medications.

Location of headache was determined using a two-stage process: First, it was noted whether in the initial consultation the pain was described as unilateral, even if only occasionally, or as bilateral. If the pain was ever unilateral, additional information was collected whether the pain was recorded as “always,” “usually,” “sometimes,” or “never” on the right side of the head, and also whether the pain was “always,” “usually,” “sometimes,” or “never” on the left side of the head.

To provide quantitative information for statistical analysis, we used a commonly used method to assess asymmetry in studies of lateralized phenomena [19]. Two laterality scores were computed for each patient. The left laterality score was determined by assigning a value of 1 if patients reported they “never” had left-sided pain, a value of 2 if they “sometimes” had left-sided pain, 3 if they “usually” had left-sided pain, and 4 if they “always” had left-sided pain. The same procedure was followed to compute the right laterality score for patients. The left score was then subtracted from the right score to produce a net numerical value reflecting headache lateralization. Thus, the range of possible values for the overall numerical score was −3 to +3, with −3 indicating pain that was always left sided (side-locked left-sided pain) and +3 indicating pain that was always right sided (side-locked right-sided pain).

To illustrate, a patient with unilateral headache who “sometimes” had pain on the left and “usually” had pain on the right would receive a left laterality score of 2 and a right laterality score of 3, for a net score of 1, indicating a slight predominance of right-sided head pain. Alternatively, a patient with unilateral headache who “always” had left-sided head pain and “never” had right-sided head pain would receive a left laterality score of 4 and a right laterality score of 1, for a net score of −3, indicating side-locked left-head pain.

In addition to examining cases in which headache was exclusively one sided, the categories of always, usually, and sometimes as well as always and usually for both left and right pain location were collapsed. This procedure made it possible to examine the association of outcomes with varying degrees of unilaterality.

Psychiatric diagnoses were identified by searching the medical record for physician diagnoses of substance use disorder, anxiety disorder, depression, bipolar disorder, cognitive/dementia disorder, eating disorder, psychotic disorder, PTSD, and/or a past history of trauma or abuse. Patients were considered to have these disorders if these diagnoses appeared in problems lists or were described in physician notes, especially the past medical history section of the record. Psychiatric diagnoses could have been assigned by any of the clinicians who saw the patient. In many cases, these were psychiatrists or psychologists, although diagnoses also could be made by primary care physicians or other healthcare providers. Psychiatric diagnoses also were inferred from prescriptions for medications principally or only used to treat psychiatric illnesses (i.e., maintenance prescriptions of any selective serotonin reuptake inhibitor or selective serotonin/norepinephrine reuptake inhibitor or antipsychotic medications). A psychiatric diagnosis was not inferred in patients using benzodiazepines or tricyclic antidepressants because those classes of medications are often used for nonpsychiatric indications.

Use of healthcare resources was measured using data from the LMR, first by determining whether a patient received primary medical care within the Partners healthcare system, as indicated by having a Partners primary care provider note listed in their medical record. Those who did not have a primary care provider within the Partners system were excluded because of inability to confirm the accuracy of their medical record or information about healthcare use. Beginning one year before the first headache consultation and up to the time of the headache consultation, the number of times patients initiated contact with a physician was counted.

Patient-initiated telephone calls, emails, appointments, urgent care visits, and procedures were counted as contacts. Laboratory and imaging reports or calls or emails initiated by healthcare providers were not included because they were not initiated by the patient. Emergency department visits were counted separately and were not included in the measures of general healthcare use. The same process was used to measure healthcare use in the year following the first headache consultation. The headache consultation itself was not counted in either the “before” or “after” contacts. In some cases, patients only had primary care notes visible in the medical record in the year following their headache consultation, indicating that they had moved their care to the Partners system. Data from these patients contributed to measures of healthcare use only for the year after consultation.

Information was collected on emergency department visits occurring in the year before and the year after the initial headache consultation visit. Visits were categorized as due to headache or for other reasons, based on information contained in physician or nursing notes. Urgent care visits were not included in these counts.

### 2.2. Statistical Analyses

Preliminary data analyses were conducted after collecting information on 50 patients during the pilot portion of the study. Power calculations indicated that a sample size of 500 patients would be sufficient to detect a difference of 20% in the proportion of patients with depression or anxiety in the group with left-lateralized pain compared with the group with right-lateralized pain. Pilot patients were not included in the final analyses.

For the actual study, demographic information was obtained and logistic regression was used to evaluate the relationship between demographic and other factors including markers of healthcare use (such as ED and general medical visits) and distress (such as depression or anxiety). All analyses were generated using SAS software v 4.3^©^ 2011, SAS Institute Inc, Cary, NC. The *p* values for the distress measurements were obtained by subtracting the right from the left score and using the Jonckheere–Terpstra test.

## 3. Results

### 3.1. Characteristics of the Entire Sample

Of the 500 patient records identified for the actual study, 2 did not contain a consultation note and were excluded from the analysis. Of the 498 nonpilot patients for whom consultation notes were available, 477 (95.8%) patients had migraine. Sixteen (3.2%) patients had cluster headache, and five (1%) patients had hemicrania continua. The 21 patients with cluster headache or hemicrania continua were removed from the final analyses of migraine patients.

### 3.2. Demography of the Migraine Population

Table 1 displays the demographic, handedness, and headache characteristics of the 477 study subjects with migraine. Four hundred twenty-four (88.9%) patients were females. The mean age was 40.4 years (range 17–83). Of the 365 patients with migraine for whom information on aura was available, 96 (26.3%) patients had migraine with aura.

Of the 91.6% of patients who provided handedness information at the initial consultation, 33/437 (7.6%) of patients were left handed. The majority of patients were Caucasian, married, with over 80% having more than a high school diploma. 13.7% of patients were unemployed or disabled. Within this sample, the majority (81.5%) of patients reported onset of headaches before the age of 26.

### 3.3. Laterality of Headache

Table 2 displays the characteristics of headache location. Headache laterality could not be determined from medical record review in 26 of 477 (5.5%) patients with migraine because of missing or ambiguous descriptions.

Among patients with migraine, 228 of the 451 (50.6%) patients with available information reported some lateralized pain, whether it was strictly side-locked pain, usually, or always lateralized. Of the 228 subjects with lateralized pain, 72 of 228 (31.6%) patients had side-locked pain, with twice as many (24 L and 48 R) reporting right side-locked pain compared with left side-locked pain. Thus, of the entire 451 subjects, only 72 (15.9%) patients had side-locked pain. When considering patients with any amount of right- or left-sided pain predominance, 107 of 228 (47%) patients had right pain predominance and 65 of 228 (28.5%) patients had a predominance of left-sided headaches (*p* < 0.001). 56 of 228 (24.5%) patients with unilateral headache had pain that was equally distributed between the right and left side of the head.  Table 3 shows the differences between frequency of side experience among the 228 migraineurs with lateralized headaches, showing that about a quarter of such patients report headaches which are evenly distributed between the right and left side of the head. Among those whose headaches are more common on one side or the other, there is a clear predominance of right-sided headaches. Twice as many patients with side-locked headaches report that headaches are right sided (48 versus 24).

### 3.4. Anxiety, Depression, and Healthcare Use

Table 4 shows the prevalence of selected psychiatric comorbidities, medication, and healthcare use in patients with migraine and their relation to headache laterality. Almost a third of patients had a diagnosis of depression recorded in or inferred from the medical record, while about 30% had anxiety. One hundred thirty-one of four hundred twenty-four (30.9%) females had anxiety compared with 13 of 53 (24.5%) males, but this difference was not statistically significant (*p*=0.34). One hundred forty-three of four hundred twenty-four (33.7%) females and fourteen of fifty-three (26.4%) males had depression, which was not a statistically significant difference (*p*=0.29).

15.2% of migraine patients visited the emergency department (ED) for headache in the year before their first consultation visit, compared with 10.4% in the year after the first consultation. ED visits for other reasons also declined. Moreover, the average number of ED visits for headache in the year prior to consultation was 0.32 (range 0–22) and in the year after consultation was 0.18 (0–6). Thirty-one of two hundred twenty-one females (14%) and seven of twenty-nine (24%) males went to the ED in the year before the first consultation, with no significant sex differences (*p*=0.15). Twenty-nine of two hundred sixty (11%) females went to the ED in the year after the first consultation, versus two of thirty-eight (5%) males, which was not statistically significant (*p*=0.27).

Tables 5 [Table tab6] [Table tab7]–8 contain detailed information on healthcare use and measures of distress and their association with headache lateralization. Except for posttraumatic stress disorder, there were no statistically significant differences between any measure of left versus right lateralization for these variables; that is, patients with left-lateralized pain, whether side locked or not, were not significantly more likely than patients with right-lateralized pain to have depression, anxiety, or higher levels of healthcare utilization such as ED or healthcare visits for headache or other reasons. For patients with information on both variables available, for example, 14 of 80 (17.5%) patients with any degree of right-sided pain predominance went to the ED in the year before their first consultation, compared with 5 of 45 (11.1%) patients with left-sided pain predominance (*p*=0.408, Pearson chi-square).

The single exception was that patients with left-lateralized pain were statistically significantly more likely than those with right-lateralized pain to have a diagnosis of posttraumatic stress disorder. Specifically, for those with information on both variables, 6 of 65 (9.2%) patients with any degree of left-predominant head pain had a history of PTSD compared with 1 of 107 with right-predominant pain (*p*=0.008, Pearson chi-square).

Bivariate correlation between ED visits for migraine in the year before the first consultation and healthcare visits in general showed a slight negative correlation that was not statistically significant. In the year after the first consultation, the number of ED visits was positively correlated with the overall number of patient-initiated healthcare contacts. (Pearson coefficient of 0.377, *p* < 0.001).

## 4. Discussion

This large study of migraine patients seen in a tertiary care specialty headache clinic demonstrates the feasibility of determining the headache location and laterality using routinely collected electronic medical record data. Information on the headache location was missing or was not interpretable in less than 6% of patients. Among this group of specialty headache providers, most initial consultation notes indicated whether head pain was lateralized, although information on the degree of lateralization was not always available.

Fewer than half of patients with migraine report that their pain is unilateral. This observation is remarkable given that migraine is commonly thought of as a lateralized pain disorder—in fact, the word migraine is derived from Latin via the Greek term hemikrania, which means “half of the cranium”. Unilateral location of pain also is a key criterion for the diagnosis of migraine. As early as 1933, the eminent neurologist Macdonald Critchley, then an assistant physician at Queen Square in London, pointed out that the headache of migraine was “often not unilateral,” characterizing this as one of the “certain incorrect or exaggerated statements commonly made.” Writing in the *Lancet*, he noted that “although this statement challenges the very name of the malady, nevertheless careful introspection reveals that in a high proportion (perhaps even 50 per cent) the pain does not strictly concern one-half the head” [20].

Only about half of patients with migraine reported unilateral pain. About a quarter of these patients say that their unilateral headaches are equally likely to occur on the right or the left. The remaining three quarters note a side preference. Among patients whose headache pain is lateralized, almost twice as many report that the pain location is more commonly or always on the right compared to the left side, and this difference is statistically significant. It is not clear whether this asymmetry is the result of hemispheric lateralization or other factors, which should be evaluated separately. This right-sided predominance is seen in many other lateralized head pain disorders and also exists for chewing-side preference. For example, the right side is more commonly involved than the left in trigeminal neuralgia and temporomandibular joint dysfunction [21, 22]. Additionally, most people prefer to chew food on the right rather than the left side of the mouth. Chewing-side preference is also correlated with other factors that reflect brain organization, such as handedness, footedness, and eye dominance [23]. This pattern of right-sided predominance in a wide variety of head pain disorders which presumably have heterogeneous causes may deserve more attention from researchers.

In general our findings that only about half of migraine patients reported unilateral pain are broadly consistent with other studies; for example, previous research did not examine asymmetry in laterality as we did using a laterality index. Rather, they simply asked patients whether headaches were typically unilateral and if so on whether there was side predominance. Lipscombe and Prior found that 66% of those with migraine had unilateral headache, and two-thirds of those reported a side preference in their headaches [24]. Compared with this, Chakravarty et al. found that, in established headache, unilateral pain was present in just 40.5% of adults and only 10.5% of children [25]. Kelman found, in a cohort of 1283 patients with migraine, that 27.3% reported headaches that were usually right sided, 24.3% reported headaches that were usually left sided, and 23.7% reported that headache was most commonly bilateral. A further 15.0% reported unilateral headaches that could be on the either side of the head. Pain was in the middle of the head in 4.6% [27].

Our hypothesis was based on the assumption that cortical activation and pain processing associated with headache pain are strongly lateralized, which could lead to differential effects depending on whether head pain arose on the right or the left. However, recent work by others, in addition to our finding of no major differences, is not consistent with this hypothesis. Other possibilities include bilateral cortical activation, or that decussation of the cranial nerves, particularly the trigeminal, may be more complex than is currently appreciated [28].

In contrast to our hypothesis that left-sided head pain would be associated with more psychopathology than right-sided pain, we found no evidence of left-sided migraine pain being more associated with psychopathology than right-sided pain. With the exception of PTSD, there was no evidence of a relationship between left-sided pain and measures of healthcare utilization or psychological distress. The statistically significant association of greater left- than right-sided head pain with PTSD may be spurious but could be explored in subsequent studies. Emergency department visits and patient-initiated contacts with the medical system declined following consultation, suggesting the value of specialty treatment for headache. There was no evidence that ED use, depression, or anxiety were more common in those with left-sided headache compared with right-sided headache.

## 5. Strengths and Limitations

A major strength of this study is the detailed and carefully documented information on a large number of patients with migraine, diagnosed by headache experts in a large, integrated healthcare system. These findings are likely to be generalizable to other clinical settings. The design and implementation of this study were based on hypotheses derived from previous work, with analyses based on a protocol developed before data collection, and sample size was determined based on a preliminary pilot study.

This study also has a number of limitations. Data were collected as part of a retrospective study of medical records and clinical notes of patients seeking treatment for migraine headaches in the context of routine clinical practice. Standardized data collection instruments were not used. Rather, physician notes included narrative descriptions of headache and other patient characteristics, which often lacked information such as standardized measures of pain intensity or duration. The completeness and quality of information varied among providers, requiring some subjectivity in classifying the degree of sidedness, although information in the chart was almost always adequate to make a valid basic distinction between pain that was predominantly right or left sided. It seems very likely, therefore, that the reported findings are valid. The presence of comorbid conditions such as depression was inferred based on the use of specific antidepressant or other medications, or on a mention of these diagnoses in the chart. Similarly, it was only possible to collect information on ED visits and healthcare contacts that occurred within the Partners Healthcare System. Because these methods are somewhat imprecise, it is possible that the hypothesized right versus left differences in the frequency of these outcomes may have been overlooked or underestimated.

Retrospective analysis of patient records is not an ideal way to make a diagnosis of psychiatric comorbidity, since an accurate measure of severity is not available.

Psychiatric comorbidity may not be a good measure of distress from a neural perspective. Psychiatric conditions are common and are of varying severity. Some patients with formal psychiatric diagnoses may not be particularly distressed, while others with important distress may not receive a psychiatric diagnosis. Even if not the best measure, however, we think it is reasonable to assume that severe distress would be associated with an increased likelihood of having a psychiatric diagnosis compared to matched controls and would be clinically important. Unfortunately, validated tools to assess headache impact, such as the Migraine Disability Assessment (MIDAS) or Headache Impact Test (HIT-6), were not routinely obtained.

We assessed ED utilization as a marker of distress but were unable to control for migraine severity or duration, or control for use of triptans or preventive medications, which may be confounders. Unfortunately, such information was not sufficient to calculate these factors. It seems reasonable, however, to assume that since all patients included in the study were seen and diagnosed by headache specialists in a tertiary care setting, they represent a relatively homogeneous population of treatment-seeking patients with migraine.

Our results, although very useful for the purposes of our study, highlight the shortcomings of information contained in medical records. In the future, a systematic way of collecting data, perhaps using structured questionnaires with data entered directly by patients, should be considered as a way to improve data quality and completeness.

## 6. Conclusions

Taken as a whole, our data do not support the view that left-sided migraine pain is more distressing than right-sided pain. Although unilateral pain location can be helpful in making a migraine diagnosis, it does not appear to have additional clinical implications. Additionally, its absence does not rule out a diagnosis of migraine since more than half of migraineurs have bilateral head pain.

## Figures and Tables

**Table 1:** Demographic characteristics of the migraine sample.

All patients with migraine (*N*=477)	*n* ^*∗*^
Female	424/477 (88.9%)
Male	53/477 (11.1%)
Average age in years	40.4 (range 17 to 83)
Left handed	33/437 (7.6%)
Right handed	399/437 (91.3%)
Right hand preference	2/437 (0.5%)
Ambidextrous	3/437 (0.7%)
Race	
White	391/418 (93.5%)
Black or African American	20/418 (4.8%)
Asian	2/418 (0.5%)
Native American	4/418 (1.0%)
Others	1/418 (0.2%)
Ethnicity	
Not Hispanic	387/419 (92.4%)
Hispanic	32/419 (7.6%)
Education	
College/graduate school	247/412 (60.0%)
Some college	92/412 (22.3%)
High school graduation	67/412 (16.3%)
Some high school	6/412 (1.5%)
Employed	253/389 (65.0%)
Unemployed	31/389 (8.0%)
Disabled	22/389 (5.7%)
Student	31/389 (8.0%)
Homemaker	38/389 (9.8)
Retired	14/389 (3.6%)

^*∗*^Denominators vary because of missing or uninterpretable information.

**Table 2:** Aura characteristics at patient presentation.

Characteristic	Number^*∗*^ (%)
Aura	96/365 (26.3%)
Age at headache onset: ≤10 years	118/443 (26.6%)
Age at headache onset: 11–25 years	242/443 (54.9%)
Age at headache onset: ≥26 years	82/443 (18.5%)

^*∗*^Denominators vary because information on aura was not recorded for all patients.

**Table 3:** Frequency of side experience among migraineurs with lateralized headache^*∗*^.

Side-locked left	Usually left sided	Sometimes left sided	Unilateral but no recalled predominance	Sometimes right sided	Usually right sided	Side-locked right
24 (10.5%)	15 (6.6%)	26 (11.4%)	56 (24.6%)	40 (17.5%)	19 (8.3%)	48 (21.1%)

^*∗*^ *p*=0.001 for any degree of right-sided lateralization versus any degree of left-sided lateralization.

**Table 4:** Prevalence of comorbidities and measures of healthcare use in 477 patients with migraine.

	*n*	*p* value
Depression	157/477 (32.9%)	0.514 (chi-square for males versus females)
Females	143/424 (33.7%)	
Males	14/53 (26.4%)	
Anxiety	144/477 (30.2%)	0.326 (chi-square for males versus females)
Females	131/424 (30.9%)	
Males	13/53 (24.5%)	
Patients visiting ED^*∗*^ for migraine in the year prior to headache consultation	38/250 (15.2%)	
Average number of ED visits for migraine in the year prior	0.32 (range 0–22)	0.15 (chi-square for males versus females)
Females	31/221 (14%)	
Males	7/29 (24%)	
Patients visiting ED for migraine in the year after headache consultation	31/298 (10.4%)	
Average number of ED visits for migraine in year after consultation	0.18 (range 0–6)	0.27 (chi-square for males versus females)
Females	29/260 (11%)	
Males	2/38 (5%)	
Patients visiting ED for other reasons in the year prior to headache consultation	38/247 (15.4%)	
Average number of ED visits for other reasons in the year prior	0.29 (range 0–9)	
Patients visiting ED for other reasons in the year after headache consultation	32/297 (10.8%)	
Average number of ED visits for other reasons in the year after consultation	0.17 (range 0–7)	

^*∗*^ED = emergency department.

**Table 5:** Demographic characteristics of patients with lateralized headaches (*n* = 228/451 or 47.8% of total migraineurs).

	Lateralized *n* (%)	Always left (−3) *n* (%)	Mostly left (−1 to −3) *n* (%)	Not lateralized *n* (%)	Mostly right (+1 to +3) *n* (%)	Always right (+3) *n* (%)	*p* value^*∗*^
*Sex*	228 (47.8)	24 (5.0)	65 (13.6)	223 (46.8)	107 (22.4)	48 (10.0)	0.3297
Female (%)	206 (90.4)	23 (98.8)	59 (90.8)	195 (87.4)	95 (88.8)	44 (91.7)	—
Male (%)	22 (9.6)	1 (4.2)	6 (9.2)	28 (12.6)	12 (11.2)	4 (8.3)	—
*Age (range)*	Mean = 41.29 (17–74)	Mean = 41.67 (25–59)	Mean = 43.75 (18–74)	Mean = 39.42 (17–83)	Mean = 40.86 (17–69)	Mean = 42.98 (18–69)	—
*Handedness*	*n* = 203	*n* = 23	*n* = 56	*n* = 215	*n* = 107	*n* = 44	—
Left handed	24 (10.5)	7 (30.4)	9 (16.1)	8 (3.7)	7 (7.4)	5 (11.4)	—
Right handed	178 (87.7)	25 (69.6)	47 (83.9)	204 (94.9)	88 (92.6)	39 (88.6)	0.0736
Right preference	0	0	0	1 (0.5)	0	0	—
Ambidextrous	1 (0.4)	0	0	2 (0.9)	0	0	—
*Marital status*	*n* = 221	*n* = 24	*n* = 93	*n* = 221	*n* = 141	*n* = 47	—
Married	138 (62.4)	17 (70.8)	42 (68.9)	112 (50.9)	83 (59.7)	30 (63.8)	0.7814
Single	66 (29.9)	5 (20.8)	12 (19.7)	82 (36.9)	43 (30.9)	10 (21.3)	—
Divorced	17 (7.7)	2 (8.3)	7 (11.5)	26 (11.7)	13 (9.4)	7 (14.9)	—
Widowed		0	0	1 (0.5)	0	0	—
*Education*	*n* = 198	*n* = 18	*n* = 52	*n* = 192	*n* = 126	*n* = 46	0.0700
College/graduate school	121 (53.1)	10 (55.6)	27 (51.9)	107 (55.7)	75 (59.5)	28 (60.9)	0.1174
Some college	43 (18.9)	4 (22.2)	11 (21.2)	46 (24.0)	31 (22.0)	11 (23.9)	—
High school graduation	33 (14.5)	4 (22.2)	14 (26.9)	34 (17.7)	18 (14.3)	8 (15.2)	—
Some high school	1 (0.5)	0	0	5 (2.6)	2 (1.6)	0	—
*Employment*	*n* = 189	*n* = 20	*n* = 51	*n* = 180	*n* = 124	*n* = 43	—
Employed	127 (67.2)	17 (85.0)	33 (64.7)	114 (63.3)	84 (67.7)	28 (65.1)	0.6424
Unemployed	13 (6.9)	0	4 (7.8)	17 (9.4)	9 (7.3)	3 (7.0)	—
Disabled	10 (5.3)	1 (4.2)	4 (7.8)	11 (6.1)	9 (7.3)	2 (4.7)	—
Student	13 (6.9)	0	2 (3.9)	16 (8.9)	10 (8.1)	4 (9.3)	—
Homemaker	16 (8.5)	2 (8.3)	5 (9.8)	19 (10.6)	9 (7.3)	4 (9.3)	—
Retired	10 (5.3)	0	3 (5.9)	3 (1.7)	3 (2.4)	2 (4.7)	—
*Ethnicity*	*n* = 202	*n* = 19	*n* = 53	*n* = 193	*n* = 107	*n* = 44	0.5453
Not Hispanic	183 (90.6)	18 (94.7)	47 (88.7)	181 (93.8)	92 (91.1)	40 (90.9)	—
Hispanic	19 (9.4)	1 (4.2)	6 (11.3)	12 (6.2)	9 (8.9)	4 (9.1)	—
*Race*	*n* = 202	*n* = 20	*n* = 54	*n* = 193	*n* = 100	*n* = 44	—
White	191 (94.6)	19 (95.0)	50 (92.6)	178 (92.2)	95 (95.0)	42 (95.5)	0.3275
Black	9 (4.5)	1 (5.0)	3 (5.6)	10 (5.2)	5 (4.7)	2 (4.5)	—
Asian	1 (0.5)	0	0	1 (0.5)	0	0	—
Native American	1 (0.5)	0	1 (1.9)	3 (1.3)	0	0	—
Others	0	0	0	1 (0.5)	0	0	—

^*∗*^For any degree of lateralization versus none.

**Table 6 tab6:** Headache characteristics and medication use in patients with lateralized headaches.

	Lateralized *n* (%)	Always left (−3) *n* (%)	Mostly left (−1 to −3) *n* (%)	Not lateralized *n* (%)	Mostly right (+1 to +3) *n* (%)	Always right (+3) *n* (%)	*p* value^*∗*^
*Age of onset*	*n* = 211	*n* = 24	*n* = 61	*n* = 212	*n* = 141	*n* = 44	—
Prepuberty (0–10)	57 (25.0)	5 (21.7)	18 (29.5)	54 (24.1)	24 (17.0)	11 (25.0)	—
Teen/young adult (11–25)	120 (56.9)	11 (47.8)	30 (49.2)	113 (50.4)	63 (44.7)	26 (59.1)	—
Adult (26 and older)	34 (16.1)	7 (30.4)	13 (21.3)	45 (20.1)	20 (14.2)	7 (14.6)	—
Migraine with aura (in the 365 patients who had aura information recorded)	50/170 (29.4)	7/18 (38.9)	14/50 (28.0)	37/174 (21.3)	24/76 (31.6)	11/34 (32.4)	*p* = 0.573
*Medication use before and after specialty headache consultation*							
Patients on opioids the year before consultation	55/228 (24.1)	8/24 (33.3)	22/65 (33.8)	46/223 (20.6)	22/107 (20.6)	11/48 (22.9)	*p* = 0.250
Patients on opioids the year after consultation	41/228 (18.0)	4/24 (16.7)	14/65 (21.5)	33/223 (14.8)	16/107 (15)	7/48 (14.6)	*p* = 0.3893
Patients on barbiturates the year before consultation	68/228 (29.8)	11/24 (45.8)	22/65 (33.8)	54/223 (24.2)	31/107 (29.0)	10/48 (20.8)	*p* = 0.360
Patients on barbiturates the year after consultation	58/228 (25.4)	5/24 (20.8)	14/65 (21.5)	39/223 (17.5)	31/107 (29.0)	12/48 (25.0)	*p* = 0.1298
Patients on triptans the year before consultation	128/228 (56.1)	15/24 (62.5)	35/65 (53.8)	95/223 (42.6)	63/107 (58.9)	28/48 (58.3)	*p* = 0.269
Patients on triptans the year after consultation	167/228 (73.2)	20/24 (83.3)	46/65 (70.8)	153/223 (68.6)	77/107 (72.0)	39/48 (81.3)	*p* = 0.736

^*∗*^Trend for degree of lateralization (right versus left), reported to 3 digits.

**Table 7 tab7:** Psychiatric comorbidities in patients with lateralized headaches.

	Lateralized *n* (%)	Always left (−3) *n* (%)	Mostly left (−1 to −3) *n* (%)	Not lateralized *n* (%)	Mostly right (+1 to +3) *n* (%)	Always right (+3) *n* (%)	*p* value^*∗*^
Substance use	4 (1.8)	0	1 (1.5)	10 (4.5)	1 (0.9)	0	0.6812
Anxiety	73 (32.0)	9 (37.5)	23 (35.4)	64 (28.7)	33 (30.8)	13 (27.1)	0.3265
Depression	69 (30.3)	5 (20.8)	19 (29.2)	77 (34.5)	33 (30.8)	11 (22.9)	0.5142
Bipolar disorder	10 (4.4)	1 (4.2)	4 (6.2)	10 (4.5)	3 (2.8)	2 (4.2)	0.7507
Eating disorder	3 (1.3)	0	0	8 (3.6)	0	0	0.5579
Psychotic disorder	1 (0.4)	0	0	1 (0.4)	1 (0.9)	1 (2.1)	0.0863
Posttraumatic stress disorder	8 (3.5)	3 (12.5)	6 (9.2)	8 (3.6)	1 (0.9)	1 (2.1)	0.0048

^*∗*^Chi-square, for any degree of lateralization, right versus left.

**Table 8:** Healthcare utilization behavior in patients with lateralized headaches.

	Lateralized *n* (%)	Always left (−3) *n* (%)	Mostly left (−1 to −3) *n* (%)	Not lateralized *n* (%)	Mostly right (+1 to +3) *n* (%)	Always right (+3) *n* (%)
Went to ED for migraine the year before 1st consultation	20/124 (15.4)	1/10 (10.0)	4/35 (11.4)	16/115 (13.9)	10/56 (17.9)	4/24 (16.7)
Went to ED for migraine the year after 1st consultation	19/144 (12.2)	2/13 (15.4)	5/41 (12.2)	12/142 (8.4)	11/68 (16.2)	5/27 (18.5)
Went to ED for other reasons the year before 1st consultation	20/124 (16.1)	3/10 (30)	7/35 (20.0)	17/115 (14.8)	7/55 (12.7)	4/23 (17.4)
Went to ED for other reasons the year after 1st consultation	19/144 (13.2)	0/13 (0)	1/41 (2.4)	19/142 (13.4)	5/67 (7.5)	2/27 (7.4)
Average number of patient-initiated interactions before the 1st consultation	10.0 (*n* = 122)	10.00 (*n* = 10)	11 (*n* = 35)	11.1 (*n* = 114)	10.33 (*n* = 55)	6.3 (*n* = 23)
Average number of patient-initiated interactions after the 1st consultation	14.14 (*n* = 145)	14.54 (*n* = 13)	14.88 (*n* = 41)	13.5 (*n* = 144)	13.44 (*n* = 68)	12.00 (*n* = 27)

ED = emergency department.

## Data Availability

All deidentified data supporting the analyses in this study are contained in the tables published with the paper.

## Supplementary Materials

Supplementary Materials Supplementary file with raw data and statistical comparisons.
