# Supplementary material for: Migraine Pain Location and Measures of Healthcare Use and Distress: An Observational Study
Source: Pain Res Manag. 2018 Jun 4;2018:6157982. doi: 10.1155/2018/6157982 (PMC6008805; doi:10.1155/2018/6157982)
Supplement: Supplementary Materials — Supplementary file with raw data and statistical comparisons. [file 6157982.f1.pdf]

Supplementary file with raw data and statistical comparisons

lateralized_headaches	RminusL	SideLoc	Frequency	Cumulative Frequency
.	.		26	26
.	-3	Left	1	27
.	-2	Left	2	29
.	-1	Left	7	36
.	0		26	62
.	1	Right	7	69
.	2	Right	3	72
.	3	Right	4	76
No	.		224	300
Yes	-3	Left	31	331
Yes	-2	Left	15	346
Yes	-1	Left	28	374
Yes	0		56	430
Yes	1	Right	41	471
Yes	2	Right	19	490
Yes	3	Right	60	550

Table of r_lat_score by l_lat_score

r_lat_score(Right laterality score)
l_lat_score(Left laterality score)

Frequency	1never	2sometimes	3usually	4always	Total
1never	0	3	15	32	50
2sometimes	9	67	31	2	109
3usually	19	39	14	1	73
4always	64	3	0	1	68
Total	92	112	60	36	300

~~~~~~~~~~~~~~~~~~~~~~~~~~~~~~~~

This is Never on one and Always on the other

Extreme Side	Frequency	Percent
Left	32	33.33
Right	64	66.67

ExtremeSide = Left

Proportion	0.3333
95% Lower Conf Limit	0.2404
95% Upper Conf Limit	0.4369

Test of H0: Proportion = 0.5 P = 0.0011

~~~~~~~~~~~~~~~~~~~~~~~~~~~~~~~~

This is Never on one and Non-Never on the other:

Occurs Side	Frequency	Percent
Left	50	35.21
Right	92	64.79

OccursSide = Left

Proportion 0.3521
95% Lower Conf Limit 0.2739
95% Upper Conf Limit 0.4366

Test of H0: Proportion = 0.5 P = 0.0004

~~~~~~
Migraines only:
~~~~~~

Table of r_lat_score by l_lat_score

r_lat_score(Right laterality score)
l_lat_score(Left laterality score)

Frequency	1never	2sometimes	3usually	4always	Total
1never	0	3	15	24	42
2sometimes	9	66	29	2	106
3usually	19	38	13	1	71
4always	50	3	0	1	54
Total	78	110	57	28	273

~~~~~~~~~~~~~~~~~~~~~~~~~~~~~~~~

This is Never on one and Always on the other

Extreme Side	Frequency	Percent
Left	24	32.43
Right	50	67.57

ExtremeSide = Left

Proportion 0.3243
95% Lower Conf Limit 0.2200
95% Upper Conf Limit 0.4432
~~~~~~~~~~~~~~~~~~~~~~~~~~~~~~~~

Test of H0: Proportion = 0.5   p =   0.0025

~~~~~~~~~~~~~~~~~~~~~~~~~~~~~~~~~~~~~~

This is Never on one and Non-Never on the other:

Occurs Side	Frequency	Percent
Left	42	35.00
Right	78	65.00

OccursSide = Left

Proportion	0.3500
95% Lower Conf Limit	0.2652
95% Upper Conf Limit	0.4424

Test of H0: Proportion = 0.5   p = 0.0010

Full tables

Table of sex by RminusL

sex(Sex)   RminusL

Frequency / Row Pct / Col Pct	-3	-2	-1	0	1	2	3	Total
Female	29	15	31	75	43	17	51	261
	11.11	5.75	11.88	28.74	16.48	6.51	19.54	
	90.63	88.24	88.57	91.46	89.58	77.27	79.69	
Male	3	2	4	7	5	5	13	39
~~~~~~~~~~~~~~~~~~~~~~~~~~~~~~~~~~~~~~

```
          |   7.69 |   5.13 |  10.26 |  17.95 |  12.82 |
12.82 |  33.33 |
          |   9.38 |  11.76 |  11.43 |   8.54 |  10.42 |
22.73 |  20.31 |
-------------+--------+--------+--------+--------+--------+---
-----+--------+
Total               32         17        35    82         48
22         64   300

Frequency Missing = 250


Table of White by RminusL

White     RminusL

Frequency|
Row Pct  |
Col Pct  |      -3|      -2|       -1|       0|        1|
     2|       3|  Total
---------+--------+--------+--------+--------+--------+-------
-+--------+
     0 |      1 |   3 |       1 |      4 |   2 |       1 |
5 |  17
          |   5.88 |  17.65 |   5.88 |  23.53 |  11.76 |
5.88 |  29.41 |
          |   3.70 |  21.43 |   3.45 |   5.56 |   4.35 |
4.76 |   8.33 |
---------+--------+--------+--------+--------+--------+-------
-+--------+
     1 |     26 |   11 |      28 |     68 |  44 |      20 |
55 | 252
          |  10.32 |   4.37 |  11.11 |  26.98 |  17.46 |
7.94 |  21.83 |
          |  96.30 |  78.57 |  96.55 |  94.44 |  95.65 |
95.24 |  91.67 |
---------+--------+--------+--------+--------+--------+-------
-+--------+
Total          27         14        29    72         46        21
     60   269

Frequency Missing = 281
```

```
Table of ethnicity by RminusL

ethnicity(Hispanic or Latino)      RminusL

Frequency|
Row Pct  |
Col Pct  |      -3|      -2|       -1|       0|        1|
     2|       3|  Total
---------+--------+--------+--------+--------+--------+-------
-+--------+
No        |     25 |     13 |       23 |     66 |  40 |      21
|     55 |     243
          |  10.29 |   5.35 |   9.47 |  27.16 |  16.46 |
8.64 |  22.63 |
          |  96.15 |  92.86 |  79.31 |  91.67 |  88.89 |
100.00 |  93.22 |
---------+--------+--------+--------+--------+--------+-------
-+--------+
Yes       |      1 |      1 |        6 |      6 |   5 |       0
|      4 |     23
          |   4.35 |   4.35 |  26.09 |  26.09 |  21.74 |
0.00 |  17.39 |
          |   3.85 |   7.14 |  20.69 |   8.33 |  11.11 |
0.00 |   6.78 |
---------+--------+--------+--------+--------+--------+-------
-+--------+
Total          26        14        29    72        45       21
     59   266

Frequency Missing = 284


Table of Married by RminusL

Married   RminusL

Frequency|
Row Pct  |
Col Pct  |      -3|      -2|       -1|       0|        1|
     2|       3|  Total
---------+--------+--------+--------+--------+--------+-------
-+--------+
     0 |     10 |   6 |  12 |     34 |  18 |      8 |     25 |
     113
```

```
          |   8.85 |   5.31 |  10.62 |  30.09 |  15.93 |
7.08 |  22.12 |
          |  31.25 |  37.50 |  37.50 |  41.98 |  37.50 |
38.10 |  39.68 |
---------+--------+--------+--------+--------+--------+-------
-+--------+
     1 |     22 |   10 |      20 |     47 |  30 |      13 |
38 | 180
          |  12.22 |   5.56 |  11.11 |  26.11 |  16.67 |
7.22 |  21.11 |
          |  68.75 |  62.50 |  62.50 |  58.02 |  62.50 |
61.90 |  60.32 |
---------+--------+--------+--------+--------+--------+-------
-+--------+
Total          32         16        32    81         48        21
     63   293

Frequency Missing = 257


Table of CollegeGrad by RminusL

CollegeGrad    RminusL

Frequency|
Row Pct  |
Col Pct  |      -3|      -2|       -1|       0|        1|
     2|       3|  Total
---------+--------+--------+--------+--------+--------+-------
-+--------+
     0 |      9 |   7 |  15 |     27 |  14 |      6 |     26 |
     104
          |   8.65 |   6.73 |  14.42 |  25.96 |  13.46 |
5.77 |  25.00 |
          |  37.50 |  58.33 |  50.00 |  39.13 |  32.56 |
33.33 |  41.94 |
---------+--------+--------+--------+--------+--------+-------
-+--------+
     1 |     15 |   5 |  15 |     42 |  29 |      12 |     36
|    154
          |   9.74 |   3.25 |   9.74 |  27.27 |  18.83 |
7.79 |  23.38 |
         |  62.50 |  41.67 |  50.00 |  60.87 |  67.44 |  66.67
|  58.06 |
```

	-3	-2	-1	0	1	2	3	Total
Total	24	12	30	69	43	18	62	258

Frequency Missing = 292

Table of Employed by RminusL

Employed / RminusL (Frequency, Row Pct, Col Pct)	-3	-2	-1	0	1	2	3	Total
0	4	6	14	24	14	4	19	85
	4.71	7.06	16.47	28.24	16.47	4.71	22.35	
	14.29	54.55	48.28	34.29	32.56	25.00	33.33	
1	24	5	15	46	29	12	38	169
	14.20	2.96	8.88	27.22	17.16	7.10	22.49	
	85.71	45.45	51.72	65.71	67.44	75.00	66.67	
Total	28	11	29	70	43	16	57	254

Frequency Missing = 296

Table of aura by RminusL

aura(Is aura present?)   RminusL

Frequency / Row Pct / Col Pct	-3	-2	-1	0	1	2	3	Total
No	11	8	17	32	21	8	23	120
	9.17	6.67	14.17	26.67	17.50	6.67	19.17	
	61.11	80.00	77.27	72.73	75.00	53.33	67.65	
Yes	7	2	5	12	7	7	11	51
	13.73	3.92	9.80	23.53	13.73	13.73	21.57	
	38.89	20.00	22.73	27.27	25.00	46.67	32.35	
Total	18	10	22	44	28	15	34	171

Frequency Missing = 379

Table of lateralized_headaches by RminusL

lateralized_headaches(Are headaches lateralized?)   RminusL

Frequency / Row Pct / Col Pct	-3	-2	-1	0	1	2	3	Total
No	0	0	0	0	0	0	0	0
	.	.	.	.	.	.	.	
	0.00	0.00	0.00	0.00	0.00	0.00	0.00	

```
---------+--------+-------+-------+-------+-------+-------
-+--------+
Yes       |     31 |     15 |      28 |     56 |  41 |      19
|     60 |     250
          |  12.40 |   6.00 |  11.20 |  22.40 |  16.40 |
7.60 |  24.00 |
          | 100.00 | 100.00 | 100.00 | 100.00 | 100.00 |
100.00 | 100.00 |
---------+--------+-------+-------+-------+-------+-------
-+--------+
Total          31        15        28    56        41    19
     60      250

Frequency Missing = 300


Table of MigraineDx by RminusL

MigraineDx     RminusL

Frequency|
Row Pct  |
Col Pct  |      -3|      -2|       -1|       0|        1|
     2|       3|  Total
---------+--------+-------+-------+-------+-------+-------
-+--------+
     0 |      8 |   0 |       2 |      2 |   1 |       0 |
14 |      27
          |  29.63 |   0.00 |   7.41 |   7.41 |   3.70 |
0.00 |  51.85 |
          |  25.00 |   0.00 |   5.71 |   2.44 |   2.08 |
0.00 |  21.88 |
---------+--------+-------+-------+-------+-------+-------
-+--------+
     1 |     24 |   17 |      33 |     80 |  47 |      22 |
50 | 273
          |   8.79 |   6.23 |  12.09 |  29.30 |  17.22 |
8.06 |  18.32 |
         |  75.00 | 100.00 |  94.29 |  97.56 |  97.92 | 100.00
|  78.13 |
---------+--------+-------+-------+-------+-------+-------
-+--------+
Total          32        17        35    82        48        22
     64   300
```

```
Frequency Missing = 250


Table of RightHanded by RminusL

RightHanded    RminusL

Frequency|
Row Pct  |
Col Pct  |      -3|      -2|       -1|       0|        1|
     2|       3|  Total
---------+--------+--------+--------+--------+--------+-------
-+--------+
     0 |      7 |      2 |      0 |     10 |      2 |      0 |
     7 |  28
         |  25.00 |   7.14 |   0.00 |  35.71 |   7.14 |
0.00 |  25.00 |
         |  25.00 |  13.33 |   0.00 |  12.99 |   4.88 |
0.00 |  11.86 |
---------+--------+--------+--------+--------+--------+-------
-+--------+
     1 |     21 |   13 |      29 |     67 |  39 |      20 |
52 | 241
         |   8.71 |   5.39 |  12.03 |  27.80 |  16.18 |
8.30 |  21.58 |
         |  75.00 |  86.67 | 100.00 |  87.01 |  95.12 |
100.00 |  88.14 |
---------+--------+--------+--------+--------+--------+-------
-+--------+
Total          28        15       29    77        41       20
     59   269

Frequency Missing = 281


Table of Substance by RminusL

Substance      RminusL

Frequency|
Row Pct  |
Col Pct  |      -3|      -2|       -1|       0|        1|
     2|       3|  Total
```

```
---------+--------+-------+-------+-------+-------+-------
-+--------+
     0 |     32 |   16 |      34 |     80 |  48 |      20 |
64 | 294
         |  10.88 |   5.44 |  11.56 |  27.21 |  16.33 |
6.80 |  21.77 |
         | 100.00 |  94.12 |  97.14 |  97.56 | 100.00 |
90.91 | 100.00 |
---------+--------+-------+-------+-------+-------+-------
-+--------+
     1 |      0 |   1 |       1 |      2 |   0 |       2 |
0 |       6
         |   0.00 |  16.67 |  16.67 |  33.33 |   0.00 |
33.33 |   0.00 |
         |   0.00 |   5.88 |   2.86 |   2.44 |   0.00 |
9.09 |   0.00 |
---------+--------+-------+-------+-------+-------+-------
-+--------+
Total          32       17    35         82       48    22
     64      300

Frequency Missing = 250


Table of Anxiety by RminusL

Anxiety   RminusL

Frequency|
Row Pct  |
Col Pct  |      -3|      -2|       -1|       0|       1|
     2|       3|  Total
---------+--------+-------+-------+-------+-------+-------
-+--------+
     0 |     22 |   11 |      22 |     58 |  33 |      15 |
48 | 209
         |  10.53 |   5.26 |  10.53 |  27.75 |  15.79 |
7.18 |  22.97 |
         |  68.75 |  64.71 |  62.86 |  70.73 |  68.75 |
68.18 |  75.00 |
---------+--------+-------+-------+-------+-------+-------
-+--------+
     1 |     10 |   6 |  13 |     24 |  15 |      7 |     16 |
     91
```

```
          |  10.99 |   6.59 |  14.29 |  26.37 |  16.48 |
7.69 |  17.58 |
          |  31.25 |  35.29 |  37.14 |  29.27 |  31.25 |
31.82 |  25.00 |
---------+--------+--------+--------+--------+--------+-------
-+--------+
Total          32         17        35    82         48        22
     64   300

Frequency Missing = 250


Table of Depression by RminusL

Depression     RminusL

Frequency|
Row Pct  |
Col Pct  |      -3|      -2|       -1|       0|        1|
     2|       3|  Total
---------+--------+--------+--------+--------+--------+-------
-+--------+
       0 |     26 |      10 |     21 |  53 |      33 |     11
|    51 | 205
          |  12.68 |   4.88 |  10.24 |  25.85 |  16.10 |
5.37 |  24.88 |
          |  81.25 |  58.82 |  60.00 |  64.63 |  68.75 |
50.00 |  79.69 |
---------+--------+--------+--------+--------+--------+-------
-+--------+
     1 |      6 |   7 |  14 |     29 |  15 |      11 |     13
|    95
          |   6.32 |   7.37 |  14.74 |  30.53 |  15.79 |
11.58 |  13.68 |
          |  18.75 |  41.18 |  40.00 |  35.37 |  31.25 |
50.00 |  20.31 |
---------+--------+--------+--------+--------+--------+-------
-+--------+
Total          32         17        35    82         48        22
     64   300

Frequency Missing = 250
```

Table of Bipolar by RminusL

Bipolar RminusL

Bipolar / Frequency / Row Pct / Col Pct	-3	-2	-1	0	1	2	3	Total
0	31	15	34	78	47	20	62	287
	10.80	5.23	11.85	27.18	16.38	6.97	21.60	
	96.88	88.24	97.14	95.12	97.92	90.91	96.88	
1	1	2	1	4	1	2	2	13
	7.69	15.38	7.69	30.77	7.69	15.38	15.38	
	3.13	11.76	2.86	4.88	2.08	9.09	3.13	
Total	32	17	35	82	48	22	64	300

Frequency Missing = 250

Table of Cognitive by RminusL

Cognitive RminusL

Cognitive / Frequency / Row Pct / Col Pct	-3	-2	-1	0	1	2	3	Total
0	32	17	35	82	48	22	64	300

```
          |  10.67 |   5.67 |  11.67 |  27.33 |  16.00 |
7.33 |  21.33 |
          | 100.00 | 100.00 | 100.00 | 100.00 | 100.00 |
100.00 | 100.00 |
---------+--------+--------+--------+--------+--------+-------
-+--------+
     1 |      0 |   0 |       0 |      0 |   0 |       0 |
0 |       0
          |      . |      . |       . |      . |   . |      . |
     . |
          |   0.00 |   0.00 |   0.00 |   0.00 |   0.00 |
0.00 |   0.00 |
---------+--------+--------+--------+--------+--------+-------
-+--------+
Total           32         17        35    82         48        22
     64      300

Frequency Missing = 250


Table of Eating by RminusL

Eating    RminusL

Frequency|
Row Pct  |
Col Pct  |      -3|      -2|       -1|       0|        1|
     2|       3|  Total
---------+--------+--------+--------+--------+--------+-------
-+--------+
     0 |     32 |   17 |      35 |     78 |  48 |      22 |
64 | 296
          |  10.81 |   5.74 |  11.82 |  26.35 |  16.22 |
7.43 |  21.62 |
          | 100.00 | 100.00 | 100.00 |  95.12 | 100.00 |
100.00 | 100.00 |
---------+--------+--------+--------+--------+--------+-------
-+--------+
     1 |      0 |   0 |       0 |      4 |   0 |       0 |
0 |       4
          |   0.00 |   0.00 |   0.00 | 100.00 |   0.00 |
0.00 |   0.00 |
          |   0.00 |   0.00 |   0.00 |   4.88 |   0.00 |
0.00 |   0.00 |
```

```
---------+--------+--------+--------+--------+--------+-------
-+--------+
Total          32         17        35     82         48        22
     64   300

Frequency Missing = 250


Table of Psychotic by RminusL

Psychotic      RminusL

Frequency|
Row Pct  |
Col Pct  |      -3|      -2|       -1|       0|        1|
     2|       3|  Total
---------+--------+--------+--------+--------+--------+-------
-+--------+
     0 |     32 |   17 |      35 |     82 |  48 |      21 |
63 |    298
         |  10.74 |   5.70 |  11.74 |  27.52 |  16.11 |
7.05 |  21.14 |
         | 100.00 | 100.00 | 100.00 | 100.00 | 100.00 |
95.45 |  98.44 |
---------+--------+--------+--------+--------+--------+-------
-+--------+
     1 |      0 |   0 |       0 |      0 |   0 |       1 |
1 |       2
         |   0.00 |   0.00 |   0.00 |   0.00 |   0.00 |
50.00 |  50.00 |
         |   0.00 |   0.00 |   0.00 |   0.00 |   0.00 |
4.55 |   1.56 |
---------+--------+--------+--------+--------+--------+-------
-+--------+
Total          32         17        35     82         48        22
     64   300

Frequency Missing = 250


Table of PTSD by RminusL

PTSD      RminusL
```

```
Frequency|
Row Pct  |
Col Pct  |      -3|      -2|       -1|       0|        1|
     2|       3|  Total
---------+--------+--------+--------+--------+--------+-------
-+--------+
     0 |     28 |   15 |      33 |     76 |  47 |      22 |
63 | 284
         |   9.86 |   5.28 |  11.62 |  26.76 |  16.55 |
7.75 |  22.18 |
      |  87.50 |  88.24 |  94.29 |  92.68 |  97.92 | 100.00 |
98.44 |
---------+--------+--------+--------+--------+--------+-------
-+--------+
     1 |      4 |   2 |       2 |      6 |   1 |       0 |
1 |  16
         |  25.00 |  12.50 |  12.50 |  37.50 |   6.25 |
0.00 |   6.25 |
         |  12.50 |  11.76 |   5.71 |   7.32 |   2.08 |
0.00 |   1.56 |
---------+--------+--------+--------+--------+--------+-------
-+--------+
Total          32         17        35    82         48        22
     64   300

Frequency Missing = 250


Table of OpiodsPrior by RminusL

OpiodsPrior    RminusL

Frequency|
Row Pct  |
Col Pct  |      -3|      -2|       -1|       0|        1|
     2|       3|  Total
---------+--------+--------+--------+--------+--------+-------
-+--------+
     0 |     24 |   13 |      25 |     71 |  40 |      18 |
50 | 241
         |   9.96 |   5.39 |  10.37 |  29.46 |  16.60 |
7.47 |  20.75 |
         |  75.00 |  76.47 |  71.43 |  86.59 |  83.33 |
81.82 |  78.13 |
```

```
---------+--------+-------+-------+-------+-------+-------
-+--------+
     1 |      8 |   4 |  10 |      11 |       8 |       4 |
14 |      59
         |  13.56 |   6.78 |  16.95 |  18.64 |  13.56 |
6.78 |  23.73 |
         |  25.00 |  23.53 |  28.57 |  13.41 |  16.67 |
18.18 |  21.88 |
---------+--------+-------+-------+-------+-------+-------
-+--------+
Total          32         17        35    82         48        22
     64   300

Frequency Missing = 250


Table of BarbituratesPrior by RminusL

BarbituratesPrior   RminusL

Frequency|
Row Pct  |
Col Pct  |      -3|      -2|       -1|       0|        1|
     2|       3|  Total
---------+--------+-------+-------+-------+-------+-------
-+--------+
     0 |     20 |   15 |      25 |     66 |  34 |      15 |
52 | 227
         |   8.81 |   6.61 |  11.01 |  29.07 |  14.98 |
6.61 |  22.91 |
         |  62.50 |  88.24 |  71.43 |  80.49 |  70.83 |
68.18 |  81.25 |
---------+--------+-------+-------+-------+-------+-------
-+--------+
     1 |     12 |   2 |  10 |      16 |  14 |       7 |     12 |
     73
         |  16.44 |   2.74 |  13.70 |  21.92 |  19.18 |
9.59 |  16.44 |
         |  37.50 |  11.76 |  28.57 |  19.51 |  29.17 |
31.82 |  18.75 |
---------+--------+-------+-------+-------+-------+-------
-+--------+
Total          32         17        35    82         48        22
     64   300
```

Frequency Missing = 250

Table of TriptansPrior by RminusL

TriptansPrior RminusL

Frequency / Row Pct / Col Pct	-3	-2	-1	0	1	2	3	Total
0	12	11	20	52	23	11	30	159
	7.55	6.92	12.58	32.70	14.47	6.92	18.87	
	37.50	64.71	57.14	63.41	47.92	50.00	46.88	
1	20	6	15	30	25	11	34	141
	14.18	4.26	10.64	21.28	17.73	7.80	24.11	
	62.50	35.29	42.86	36.59	52.08	50.00	53.13	
Total	32	17	35	82	48	22	64	300

Frequency Missing = 250

Table of OpioidsPost by RminusL

OpioidsPost RminusL

Frequency / Row Pct / Col Pct	-3	-2	-1	0	1	2	3	Total

```
---------+--------+-------+-------+-------+-------+-------
-+--------+
     0 |     27 |   14 |      25 |     67 |  40 |      17 |
54 | 244
         |  11.07 |   5.74 |  10.25 |  27.46 |  16.39 |
6.97 |  22.13 |
         |  84.38 |  82.35 |  71.43 |  81.71 |  83.33 |
77.27 |  84.38 |
---------+--------+-------+-------+-------+-------+-------
-+--------+
     1 |      5 |   3 |  10 |     15 |       8 |       5 |
10 |      56
         |   8.93 |   5.36 |  17.86 |  26.79 |  14.29 |
8.93 |  17.86 |
         |  15.63 |  17.65 |  28.57 |  18.29 |  16.67 |
22.73 |  15.63 |
---------+--------+-------+-------+-------+-------+-------
-+--------+
Total          32         17        35    82         48        22
     64   300

Frequency Missing = 250


Table of BarbituratesPost by RminusL

BarbituratesPost    RminusL

Frequency|
Row Pct  |
Col Pct  |      -3|      -2|       -1|       0|        1|
     2|       3|  Total
---------+--------+-------+-------+-------+-------+-------
-+--------+
     0 |     26 |   15 |      26 |     66 |  34 |      14 |
50 | 231
         |  11.26 |   6.49 |  11.26 |  28.57 |  14.72 |
6.06 |  21.65 |
         |  81.25 |  88.24 |  74.29 |  80.49 |  70.83 |
63.64 |  78.13 |
---------+--------+-------+-------+-------+-------+-------
-+--------+
     1 |      6 |   2 |       9 |     16 |   14 |      8 |
14 |      69
```

```
         |   8.70 |   2.90 |  13.04 |  23.19 |  20.29 |
11.59 |  20.29 |
         |  18.75 |  11.76 |  25.71 |  19.51 |  29.17 |
36.36 |  21.88 |
---------+--------+--------+--------+--------+--------+-------
-+--------+
Total          32        17        35    82        48       22
     64   300

Frequency Missing = 250


Table of TriptansPost by RminusL

TriptansPost   RminusL

Frequency|
Row Pct  |
Col Pct  |      -3|      -2|       -1|       0|        1|
     2|       3|  Total
---------+--------+--------+--------+--------+--------+-------
-+--------+
     0 |      8 |   5 |  13 |      25 |  14 |       9 |     14 |
     88
         |   9.09 |   5.68 |  14.77 |  28.41 |  15.91 |
10.23 |  15.91 |
         |  25.00 |  29.41 |  37.14 |  30.49 |  29.17 |
40.91 |  21.88 |
---------+--------+--------+--------+--------+--------+-------
-+--------+
     1 |     24 |   12 |      22 |      57 |  34 |      13 |
50 | 212
         |  11.32 |   5.66 |  10.38 |  26.89 |  16.04 |
6.13 |  23.58 |
         |  75.00 |  70.59 |  62.86 |  69.51 |  70.83 |
59.09 |  78.13 |
---------+--------+--------+--------+--------+--------+-------
-+--------+
Total          32        17        35    82        48       22
     64   300

Frequency Missing = 250
```

Table of race by RminusL

race(Race) RminusL

race(Race)	Statistic	-3	-2	-1	0	1	2	3	Total
American Indian or Alaska Native	Frequency	0	1	0	0	0	0	0	1
	Row Pct	0.00	100.00	0.00	0.00	0.00	0.00	0.00	
	Col Pct	0.00	7.14	0.00	0.00	0.00	0.00	0.00	
Asian	Frequency	0	0	1	2	0	0	1	4
	Row Pct	0.00	0.00	25.00	50.00	0.00	0.00	25.00	
	Col Pct	0.00	0.00	3.45	2.78	0.00	0.00	1.67	
Black or African /American	Frequency	1	2	0	2	2	1	4	12
	Row Pct	8.33	16.67	0.00	16.67	16.67	8.33	33.33	
	Col Pct	3.70	14.29	0.00	2.78	4.35	4.76	6.67	
White	Frequency	26	11	28	68	44	20	55	252
	Row Pct	10.32	4.37	11.11	26.98	17.46	7.94	21.83	
	Col Pct	96.30	78.57	96.55	94.44	95.65	95.24	91.67	
Other	Frequency	0	0	0	0	0	0	0	0

```
               |    . |      . |   . |      . |      . |   .
|    . |
               |   0.00 |   0.00 |   0.00 |   0.00 |   0.00 |
0.00 |   0.00 |
-----------------+--------+--------+--------+--------+--------
+--------+--------+
Total               27        14        29    72        46
21        60   269

Frequency Missing = 281


Table of marital_status by RminusL

marital_status(Marital Status)     RminusL

Frequency|
Row Pct  |
Col Pct  |      -3|      -2|       -1|       0|        1|
     2|       3|  Total
---------+--------+--------+--------+--------+--------+-------
-+--------+
Married  |     22 |      10 |      20 |     47 |  30 |      13
|     38 |     180
         |  12.22 |   5.56 |  11.11 |  26.11 |  16.67 |
7.22 |  21.11 |
         |  68.75 |  62.50 |  62.50 |  58.02 |  62.50 |
61.90 |  60.32 |
---------+--------+--------+--------+--------+--------+-------
-+--------+
Single   |      5 |      4 |       7 |     30 |   16 |      7
|     17 |     86
         |   5.81 |   4.65 |   8.14 |  34.88 |  18.60 |
8.14 |  19.77 |
         |  15.63 |  25.00 |  21.88 |  37.04 |  33.33 |
33.33 |  26.98 |
---------+--------+--------+--------+--------+--------+-------
-+--------+
Divorced |     4 |      1 |   5 |       4 |      2 |   1 |
     8 |     25
         |  16.00 |   4.00 |  20.00 |  16.00 |   8.00 |
4.00 |  32.00 |
         |  12.50 |   6.25 |  15.63 |   4.94 |   4.17 |
4.76 |  12.70 |
```

```
---------+--------+-------+--------+--------+--------+-------
-+--------+
Widowed  |      1 |      0 |        0 |      0 |   0 |        0
|      0 |     1
         | 100.00 |   0.00 |   0.00 |   0.00 |   0.00 |
0.00 |   0.00 |
         |   3.13 |   0.00 |   0.00 |   0.00 |   0.00 |
0.00 |   0.00 |
---------+--------+-------+--------+--------+--------+-------
-+--------+
Other     |      0 |      1 |        0 |      0 |   0 |        0
|      0 |     1
         |   0.00 | 100.00 |   0.00 |   0.00 |   0.00 |
0.00 |   0.00 |
         |   0.00 |   6.25 |   0.00 |   0.00 |   0.00 |
0.00 |   0.00 |
---------+--------+-------+--------+--------+--------+-------
-+--------+
Total          32         16        32    81         48        21
     63   293

Frequency Missing = 257


Table of education by RminusL

education(Education)     RminusL

Frequency      |
Row Pct        |
Col Pct        |      -3|     -2|       -1|       0|        1|
     2|       3|  Total
----------------+--------+--------+--------+--------+--------
+--------+--------+
Some high school |       0 |       0 |      0 |   3 |       0
|      0 |     1 |       4
 or less         |   0.00 |   0.00 |   0.00 |  75.00 |   0.00
|   0.00 |  25.00 |
               |   0.00 |   0.00 |   0.00 |   4.35 |   0.00 |
0.00 |   1.61 |
----------------+--------+--------+--------+--------+--------
+--------+--------+
High school grad |       5 |       5 |      8 |   5 |       7
|      2 |     8 |  40
```

```
uate or GED     |  12.50 |  12.50 |  20.00 |  12.50 |  17.50 |
5.00 |  20.00 |
                |  20.83 |  41.67 |  26.67 |   7.25 |  16.28 |
11.11 |  12.90 |
-----------------+--------+--------+--------+--------+--------
+--------+--------+
Some college    |      4 |      2 |       7 |     19 |   7 |
     4 |     17 |   60
                |   6.67 |   3.33 |  11.67 |  31.67 |  11.67 |
6.67 |  28.33 |
                |  16.67 |  16.67 |  23.33 |  27.54 |  16.28 |
22.22 |  27.42 |
-----------------+--------+--------+--------+--------+--------
+--------+--------+
Graduate or coll |  15 |      5 |     15 |   42 |      29 |
12 |      36 | 154
ege or postgradu |   9.74 |   3.25 |   9.74 |  27.27 |  18.83
|   7.79 |  23.38 |
ate degree      |  62.50 |  41.67 |  50.00 |  60.87 |  67.44 |
66.67 |  58.06 |
-----------------+--------+--------+--------+--------+--------
+--------+--------+
Total               24         12        30    69         43
18        62   258

Frequency Missing = 292


Table of employment by RminusL

employment(Employment status)      RminusL

Frequency  |
Row Pct    |
Col Pct   |      -3|     -2|       -1|       0|        1|
     2|       3|  Total
-----------+--------+--------+--------+--------+--------+-----
---+--------+
Employed   |     24 |    5 |  15 |     46 |  29 |      12 |
38 | 169
           |  14.20 |   2.96 |   8.88 |  27.22 |  17.16 |
7.10 |  22.49 |
          |  85.71 |  45.45 |  51.72 |  65.71 |  67.44 |
75.00 |  66.67 |
```

Unemployed	0	1	5	6	3	1	3	19
	0.00	5.26	26.32	31.58	15.79	5.26	15.79	
	0.00	9.09	17.24	8.57	6.98	6.25	5.26	
Disabled	1	1	4	3	2	1	4	16
	6.25	6.25	25.00	18.75	12.50	6.25	25.00	
	3.57	9.09	13.79	4.29	4.65	6.25	7.02	
Student	0	1	1	5	3	1	6	17
	0.00	5.88	5.88	29.41	17.65	5.88	35.29	
	0.00	9.09	3.45	7.14	6.98	6.25	10.53	
Homemaker	3	1	2	7	3	1	4	21
	14.29	4.76	9.52	33.33	14.29	4.76	19.05	
	10.71	9.09	6.90	10.00	6.98	6.25	7.02	
Retired	0	2	2	3	3	0	2	12
	0.00	16.67	16.67	25.00	25.00	0.00	16.67	
	0.00	18.18	6.90	4.29	6.98	0.00	3.51	
Total	28	11	29	70	43	16	57	254

Frequency Missing = 296

Table of headache_dx by RminusL

headache_dx(Principle Headache Diagnosis)   RminusL

Frequency / Row Pct / Col Pct	-3	-2	-1	0	1	2	3	Total
Migraine	24	17	33	80	47	22	50	273
	8.79	6.23	12.09	29.30	17.22	8.06	18.32	
	75.00	100.00	94.29	97.56	97.92	100.00	78.13	
Cluster Headache	5	0	2	2	1	0	12	22
	22.73	0.00	9.09	9.09	4.55	0.00	54.55	
	15.63	0.00	5.71	2.44	2.08	0.00	18.75	
Hemicrania continua	3	0	0	0	0	0	2	5
	60.00	0.00	0.00	0.00	0.00	0.00	40.00	
	9.38	0.00	0.00	0.00	0.00	0.00	3.13	
Total	32	17	35	82	48	22	64	300

Frequency Missing = 250

Table of hand by RminusL

hand(Handedness) RminusL

Frequency / Row Pct / Col Pct	-3	-2	-1	0	1	2	3	Total
Left	7	2	0	9	2	0	7	27
	25.93	7.41	0.00	33.33	7.41	0.00	25.93	
	25.00	13.33	0.00	11.69	4.88	0.00	11.86	
Right	21	13	29	67	39	20	52	241
	8.71	5.39	12.03	27.80	16.18	8.30	21.58	
	75.00	86.67	100.00	87.01	95.12	100.00	88.14	
Right preference	0	0	0	0	0	0	0	0
	.	.	.	.	.	.	.	
	0.00	0.00	0.00	0.00	0.00	0.00	0.00	
Ambidextrous	0	0	0	1	0	0	0	1
	0.00	0.00	0.00	100.00	0.00	0.00	0.00	
	0.00	0.00	0.00	1.30	0.00	0.00	0.00	
Total	28	15	29	77	41	20	59	269

Frequency Missing = 281

~~~~~~

Jonckheere-Terpstra Test for trend p-values for the binary variables with RminusL

Table sex * RminusL	0.0483
Table White * RminusL	0.9163
Table ethnicity * RminusL	0.3976
Table Married * RminusL	0.6294
Table CollegeGrad * RminusL	0.4825
Table Employed * RminusL	0.9359
Table aura * RminusL	0.5733
Table MigraineDx * RminusL	0.3394
Table RightHanded * RminusL	0.2119
Table Substance * RminusL	0.6522
Table Anxiety * RminusL	0.3231
Table Depression * RminusL	0.4610
Table Bipolar * RminusL	0.7127
Table Eating * RminusL	0.5542
Table Psychotic * RminusL	0.1076
Table PTSD * RminusL	0.0058
Table OpiodsPrior * RminusL	0.5525
Table BarbituratesPrior * RminusL	0.3751
Table TriptansPrior * RminusL	0.4989
Table OpioidsPost * RminusL	0.5615
Table BarbituratesPost * RminusL	0.3041
Table TriptansPost * RminusL	0.5092

Correlation of education and RminusL is 0.06506, p=0.2979, n=258.

The analyses below help to see the direction of the PTSD trend:

Analysis Variable : RminusL Left would be more negative and right would be more positive:

PTSD	Obs	Mean	Std Dev	N	Median	Minimum	Maximum
~~~~~~

```
          0    521        0.4683099       1.8653572    284
     0    -3.0000000     3.0000000
          1     29       -0.8750000     1.7078251      16
-0.5000000     -3.0000000     3.0000000
--------------------------------------------------------------
--------------------------------------------
```

PTSD sufferers differ from nonPTSD individuals whereby sufferers are on the left side on average, and non-sufferers are slightly on the right. Same can be seen in the crosstabulation above (p<0.01). Note that the N with PTSD is only 16.

~~~~~~

MIGRAINE DX only

~~~~~~

```
Table of sex by RminusL

sex(Sex)       RminusL

Frequency |
Row Pct   |
Col Pct   |      -3|     -2|       -1|       0|        1|
     2|       3|  Total
-------------+--------+--------+--------+--------+--------+---
-----+--------+
Female    |     23 |     15 |      30 |     73 |  43 |      17
|     45 |     246
          |   9.35 |   6.10 |  12.20 |  29.67 |  17.48 |
6.91 |  18.29 |
          |  95.83 |  88.24 |  90.91 |  91.25 |  91.49 |
77.27 |  90.00 |
```

```
-------------+--------+--------+--------+--------+--------+---
-----+--------+
Male      |      1 |      2 |       3 |      7 |   4 |       5
|      5 |     27
          |   3.70 |   7.41 |  11.11 |  25.93 |  14.81 |
18.52 |  18.52 |
          |   4.17 |  11.76 |   9.09 |   8.75 |   8.51 |
22.73 |  10.00 |
-------------+--------+--------+--------+--------+--------+---
-----+--------+
Total               24         17        33    80         47
22        50   273

Frequency Missing = 250


Table of White by RminusL

White     RminusL

Frequency|
Row Pct  |
Col Pct  |      -3|      -2|       -1|       0|       1|
     2|       3|  Total
---------+--------+--------+--------+--------+--------+-------
-+--------+
     0 |      1 |   3 |       1 |      4 |   2 |      1 |   2
|    14
          |   7.14 |  21.43 |   7.14 |  28.57 |  14.29 |
7.14 |  14.29 |
          |   5.00 |  21.43 |   3.70 |   5.71 |   4.44 |
4.76 |   4.35 |
---------+--------+--------+--------+--------+--------+-------
-+--------+
     1 |     19 |   11 |      26 |     66 |  43 |      20 |
44 | 229
          |   8.30 |   4.80 |  11.35 |  28.82 |  18.78 |
8.73 |  19.21 |
          |  95.00 |  78.57 |  96.30 |  94.29 |  95.56 |
95.24 |  95.65 |
---------+--------+--------+--------+--------+--------+-------
-+--------+
Total          20         14        27    70         45        21
     46   243
```

Frequency Missing = 280

Table of ethnicity by RminusL

ethnicity(Hispanic or Latino) RminusL

Frequency / Row Pct / Col Pct	-3	-2	-1	0	1	2	3	Total
No	18	13	22	64	40	21	42	220
	8.18	5.91	10.00	29.09	18.18	9.55	19.09	
	94.74	92.86	81.48	91.43	88.89	100.00	91.30	
Yes	1	1	5	6	5	0	4	22
	4.55	4.55	22.73	27.27	22.73	0.00	18.18	
	5.26	7.14	18.52	8.57	11.11	0.00	8.70	
Total	19	14	27	70	45	21	46	242

Frequency Missing = 281

Table of Married by RminusL

Married RminusL

Frequency / Row Pct / Col Pct	-3	-2	-1	0	1	2	3	Total

```
---------+--------+-------+-------+--------+--------+-------
-+--------+
     0 |      7 |   6 |  11 |     33 |  18 |      8 |     18 |
     101
          |   6.93 |   5.94 |  10.89 |  32.67 |  17.82 |
7.92 |  17.82 |
          |  29.17 |  37.50 |  36.67 |  41.77 |  38.30 |
38.10 |  36.73 |
---------+--------+-------+-------+--------+--------+-------
-+--------+
     1 |     17 |   10 |      19 |     46 |  29 |      13 |
31 | 165
          |  10.30 |   6.06 |  11.52 |  27.88 |  17.58 |
7.88 |  18.79 |
          |  70.83 |  62.50 |  63.33 |  58.23 |  61.70 |
61.90 |  63.27 |
---------+--------+-------+-------+--------+--------+-------
-+--------+
Total          24         16        30    79        47       21
     49   266

Frequency Missing = 257


Table of CollegeGrad by RminusL

CollegeGrad    RminusL

Frequency|
Row Pct  |
Col Pct  |      -3|      -2|       -1|       0|        1|
     2|       3|  Total
---------+--------+-------+-------+--------+--------+-------
-+--------+
       0 |     8 |        7 |     14 |   27 |      14 |      6
|    18 |      94
          |   8.51 |   7.45 |  14.89 |  28.72 |  14.89 |
6.38 |  19.15 |
          |  44.44 |  58.33 |  50.00 |  40.30 |  32.56 |
33.33 |  37.50 |
---------+--------+-------+-------+--------+--------+-------
-+--------+
     1 |     10 |   5 |  14 |     40 |  29 |      12 |     30
|    140
```

```
         |   7.14 |   3.57 |  10.00 |  28.57 |  20.71 |
8.57 |  21.43 |
         |  55.56 |  41.67 |  50.00 |  59.70 |  67.44 |
66.67 |  62.50 |
---------+--------+--------+--------+--------+--------+-------
-+--------+
Total          18        12        28    67        43       18
     48   234

Frequency Missing = 289


Table of Employed by RminusL

Employed  RminusL

Frequency|
Row Pct  |
Col Pct  |      -3|      -2|       -1|       0|        1|
     2|       3|  Total
---------+--------+--------+--------+--------+--------+-------
-+--------+
     0 |      3 |   6 |  13 |     24 |  14 |      4 |     15 |
     79
         |   3.80 |   7.59 |  16.46 |  30.38 |  17.72 |   5.06
|  18.99 |
         |  15.00 |  54.55 |  48.15 |  35.29 |  33.33 |
25.00 |  33.33 |
---------+--------+--------+--------+--------+--------+-------
-+--------+
     1 |     17 |   5 |  14 |     44 |  28 |      12 |     30
|    150
         |  11.33 |   3.33 |   9.33 |  29.33 |  18.67 |
8.00 |  20.00 |
         |  85.00 |  45.45 |  51.85 |  64.71 |  66.67 |
75.00 |  66.67 |
---------+--------+--------+--------+--------+--------+-------
-+--------+
Total          20        11        27    68        42       16
     45   229

Frequency Missing = 294
```

Table of aura by RminusL

aura(Is aura present?) RminusL

Frequency / Row Pct / Col Pct	-3	-2	-1	0	1	2	3	Total
No	11	8	17	32	21	8	23	120
	9.17	6.67	14.17	26.67	17.50	6.67	19.17	
	61.11	80.00	77.27	72.73	75.00	53.33	67.65	
Yes	7	2	5	12	7	7	11	51
	13.73	3.92	9.80	23.53	13.73	13.73	21.57	
	38.89	20.00	22.73	27.27	25.00	46.67	32.35	
Total	18	10	22	44	28	15	34	171

Frequency Missing = 352

Table of lateralized_headaches by RminusL

lateralized_headaches(Are headaches lateralized?) RminusL

Frequency / Row Pct / Col Pct	-3	-2	-1	0	1	2	3	Total
No	0	0	0	0	0	0	0	0

```
          |      . |      . |       . |      . |   . |       .
|      . |
          |   0.00 |   0.00 |   0.00 |   0.00 |   0.00 |
0.00 |   0.00 |
---------+--------+--------+--------+--------+--------+-------
-+--------+
Yes       |     24 |     15 |      26 |     56 |  41 |      19
|     48 |     229
          |  10.48 |   6.55 |  11.35 |  24.45 |  17.90 |
8.30 |  20.96 |
          | 100.00 | 100.00 | 100.00 | 100.00 | 100.00 |
100.00 | 100.00 |
---------+--------+--------+--------+--------+--------+-------
-+--------+
Total          24        15        26    56        41       19
     48   229

Frequency Missing = 294


Table of MigraineDx by RminusL

MigraineDx     RminusL

Frequency|
Row Pct  |
Col Pct  |      -3|      -2|       -1|      0|        1|
     2|       3|  Total
---------+--------+--------+--------+--------+--------+-------
-+--------+
       1 |     24 |      17 |     33 |  80 |      47 |     22
|    50 | 273
          |   8.79 |   6.23 |  12.09 |  29.30 |  17.22 |
8.06 |  18.32 |
          | 100.00 | 100.00 | 100.00 | 100.00 | 100.00 |
100.00 | 100.00 |
---------+--------+--------+--------+--------+--------+-------
-+--------+
Total          24        17        33    80        47       22
     50   273

Frequency Missing = 250
```

Table of RightHanded by RminusL

RightHanded RminusL

Frequency / Row Pct / Col Pct	-3	-2	-1	0	1	2	3	Total
0	7	2	0	10	2	0	5	26
	26.92	7.69	0.00	38.46	7.69	0.00	19.23	
	30.43	13.33	0.00	13.33	4.88	0.00	10.87	
1	16	13	27	65	39	20	41	221
	7.24	5.88	12.22	29.41	17.65	9.05	18.55	
	69.57	86.67	100.00	86.67	95.12	100.00	89.13	
Total	23	15	27	75	41	20	46	247

Frequency Missing = 276

Table of Substance by RminusL

Substance RminusL

Frequency / Row Pct / Col Pct	-3	-2	-1	0	1	2	3	Total
0	24	16	32	78	47	20	50	267

```
          |   8.99 |   5.99 |  11.99 |  29.21 |  17.60 |
7.49 |  18.73 |
          | 100.00 |  94.12 |  96.97 |  97.50 | 100.00 |
90.91 | 100.00 |
---------+--------+--------+--------+--------+--------+-------
-+--------+
     1 |      0 |   1 |       1 |      2 |   0 |       2 |
0 |       6
          |   0.00 |  16.67 |  16.67 |  33.33 |   0.00 |
33.33 |   0.00 |
         |   0.00 |   5.88 |   3.03 |   2.50 |   0.00 |   9.09
|   0.00 |
---------+--------+--------+--------+--------+--------+-------
-+--------+
Total          24         17        33    80         47        22
     50   273

Frequency Missing = 250


Table of Anxiety by RminusL

Anxiety   RminusL

Frequency|
Row Pct  |
Col Pct  |      -3|      -2|       -1|       0|        1|
     2|       3|  Total
---------+--------+--------+--------+--------+--------+-------
-+--------+
     0 |     15 |   11 |     20 |  57 |      32 |     15 |  36
|    186
          |   8.06 |   5.91 |  10.75 |  30.65 |  17.20 |
8.06 |  19.35 |
          |  62.50 |  64.71 |  60.61 |  71.25 |  68.09 |
68.18 |  72.00 |
---------+--------+--------+--------+--------+--------+-------
-+--------+
     1 |      9 |   6 |  13 |     23 |  15 |      7 |     14 |
     87
          |  10.34 |   6.90 |  14.94 |  26.44 |  17.24 |
8.05 |  16.09 |
          |  37.50 |  35.29 |  39.39 |  28.75 |  31.91 |
31.82 |  28.00 |
```

```
---------+--------+-------+-------+-------+-------+-------
-+--------+
Total          24       17       33    80        47       22
     50   273

Frequency Missing = 250


Table of Depression by RminusL

Depression     RminusL

Frequency|
Row Pct  |
Col Pct  |      -3|      -2|       -1|       0|        1|
     2|       3|  Total
---------+--------+-------+-------+-------+-------+-------
-+--------+
     0 |     19 |   10 |      19 |     53 |  32 |      11 |
39 | 183
         |  10.38 |   5.46 |  10.38 |  28.96 |  17.49 |
6.01 |  21.31 |
         |  79.17 |  58.82 |  57.58 |  66.25 |  68.09 |
50.00 |  78.00 |
---------+--------+-------+-------+-------+-------+-------
-+--------+
     1 |      5 |   7 |  14 |     27 |  15 |      11 |     11
|    90
         |   5.56 |   7.78 |  15.56 |  30.00 |  16.67 |
12.22 |  12.22 |
         |  20.83 |  41.18 |  42.42 |  33.75 |  31.91 |
50.00 |  22.00 |
---------+--------+-------+-------+-------+-------+-------
-+--------+
Total           24       17         33        80       47    22
     50      273

Frequency Missing = 250


Table of Bipolar by RminusL

Bipolar   RminusL
```

```
Frequency|
Row Pct  |
Col Pct  |      -3|      -2|       -1|       0|        1|
     2|       3|  Total
---------+--------+--------+--------+--------+--------+-------
-+--------+
     0 |     23 |   15 |      32 |     76 |  46 |      20 |
48 | 260
         |   8.85 |   5.77 |  12.31 |  29.23 |  17.69 |
7.69 |  18.46 |
         |  95.83 |  88.24 |  96.97 |  95.00 |  97.87 |
90.91 |  96.00 |
---------+--------+--------+--------+--------+--------+-------
-+--------+
     1 |      1 |   2 |       1 |      4 |   1 |       2 |
2 |  13
         |   7.69 |  15.38 |   7.69 |  30.77 |   7.69 |
15.38 |  15.38 |
         |   4.17 |  11.76 |   3.03 |   5.00 |   2.13 |
9.09 |   4.00 |
---------+--------+--------+--------+--------+--------+-------
-+--------+
Total          24         17        33    80         47        22
     50   273

Frequency Missing = 250


Table of Cognitive by RminusL

Cognitive      RminusL

Frequency|
Row Pct  |
Col Pct  |      -3|      -2|       -1|       0|        1|
     2|       3|  Total
---------+--------+--------+--------+--------+--------+-------
-+--------+
     0 |     24 |   17 |     33 |  80 |      47 |     22 |  50
|    273
         |   8.79 |   6.23 |  12.09 |  29.30 |  17.22 |
8.06 |  18.32 |
         | 100.00 | 100.00 | 100.00 | 100.00 | 100.00 |
100.00 | 100.00 |
```

```
---------+--------+-------+--------+--------+-------+-------
-+--------+
     1 |      0 |   0 |       0 |      0 |   0 |       0 |
0 |       0
         |      . |     . |       . |      . |   . |       .
|      . |
         |   0.00 |   0.00 |   0.00 |   0.00 |   0.00 |
0.00 |   0.00 |
---------+--------+-------+--------+--------+-------+-------
-+--------+
Total          24        17        33    80         47       22
     50   273

Frequency Missing = 250


Table of Eating by RminusL

Eating    RminusL

Frequency|
Row Pct  |
Col Pct  |      -3|      -2|       -1|       0|        1|
     2|       3|  Total
---------+--------+-------+--------+--------+-------+-------
-+--------+
     0 |     24 |   17 |      33 |     76 |  47 |      22 |
50 | 269
         |   8.92 |   6.32 |  12.27 |  28.25 |  17.47 |
8.18 |  18.59 |
         | 100.00 | 100.00 | 100.00 |  95.00 | 100.00 |
100.00 | 100.00 |
---------+--------+-------+--------+--------+-------+-------
-+--------+
     1 |      0 |   0 |       0 |      4 |   0 |       0 |
0 |       4
         |   0.00 |   0.00 |   0.00 | 100.00 |   0.00 |
0.00 |   0.00 |
         |   0.00 |   0.00 |   0.00 |   5.00 |   0.00 |
0.00 |   0.00 |
---------+--------+-------+--------+--------+-------+-------
-+--------+
Total          24        17        33    80         47       22
     50   273
```

Frequency Missing = 250

Table of Psychotic by RminusL

Psychotic RminusL

Psychotic (Frequency / Row Pct / Col Pct)	-3	-2	-1	0	1	2	3	Total
0	24	17	33	80	47	21	49	271
	8.86	6.27	12.18	29.52	17.34	7.75	18.08	
	100.00	100.00	100.00	100.00	100.00	95.45	98.00	
1	0	0	0	0	0	1	1	2
	0.00	0.00	0.00	0.00	0.00	50.00	50.00	
	0.00	0.00	0.00	0.00	0.00	4.55	2.00	
Total	24	17	33	80	47	22	50	273

Frequency Missing = 250

Table of PTSD by RminusL

PTSD RminusL

PTSD (Frequency / Row Pct / Col Pct)	-3	-2	-1	0	1	2	3	Total

```
---------+--------+-------+-------+-------+-------+-------
-+--------+
     0 |     20 |   15 |      31 |     74 |  46 |      22 |
49 | 257
         |   7.78 |   5.84 |  12.06 |  28.79 |  17.90 |
8.56 |  19.07 |
         |  83.33 |  88.24 |  93.94 |  92.50 |  97.87 |
100.00 |  98.00 |
---------+--------+-------+-------+-------+-------+-------
-+--------+
     1 |      4 |   2 |       2 |      6 |   1 |       0 |
1 |  16
         |  25.00 |  12.50 |  12.50 |  37.50 |   6.25 |
0.00 |   6.25 |
         |  16.67 |  11.76 |   6.06 |   7.50 |   2.13 |
0.00 |   2.00 |
---------+--------+-------+-------+-------+-------+-------
-+--------+
Total          24         17        33    80         47        22
     50   273

Frequency Missing = 250


Table of OpiodsPrior by RminusL

OpiodsPrior    RminusL

Frequency|
Row Pct  |
Col Pct  |      -3|      -2|       -1|       0|        1|
     2|       3|  Total
---------+--------+-------+-------+-------+-------+-------
-+--------+
     0 |     16 |   13 |      23 |     69 |  39 |      18 |
39 | 217
         |   7.37 |   5.99 |  10.60 |  31.80 |  17.97 |
8.29 |  17.97 |
         |  66.67 |  76.47 |  69.70 |  86.25 |  82.98 |
81.82 |  78.00 |
---------+--------+-------+-------+-------+-------+-------
-+--------+
     1 |      8 |   4 |  10 |     11 |      8 |   4 |  11 |
56
```

```
          |  14.29 |   7.14 |  17.86 |  19.64 |  14.29 |
7.14 |  19.64 |
          |  33.33 |  23.53 |  30.30 |  13.75 |  17.02 |
18.18 |  22.00 |
---------+--------+--------+--------+--------+--------+-------
-+--------+
Total          24         17        33    80         47        22
     50   273

Frequency Missing = 250


Table of BarbituratesPrior by RminusL

BarbituratesPrior   RminusL

Frequency|
Row Pct  |
Col Pct  |      -3|      -2|       -1|       0|       1|    2|
3|  Total
---------+--------+--------+--------+--------+--------+-------
-+--------+
     0 |     13 |   15 |      23 |     64 |  33 |      15 |
40 | 203
          |   6.40 |   7.39 |  11.33 |  31.53 |  16.26 |
7.39 |  19.70 |
          |  54.17 |  88.24 |  69.70 |  80.00 |  70.21 |
68.18 |  80.00 |
---------+--------+--------+--------+--------+--------+-------
-+--------+
     1 |     11 |   2 |  10 |     16 |  14 |      7 |     10 |
     70
          |  15.71 |   2.86 |  14.29 |  22.86 |  20.00 |
10.00 |  14.29 |
          |  45.83 |  11.76 |  30.30 |  20.00 |  29.79 |
31.82 |  20.00 |
---------+--------+--------+--------+--------+--------+-------
-+--------+
Total          24         17        33    80         47        22
50   273

Frequency Missing = 250
```

Table of TriptansPrior by RminusL

TriptansPrior RminusL

TriptansPrior	Frequency / Row Pct / Col Pct	-3	-2	-1	0	1	2	3	Total
0	Frequency	9	11	19	50	22	11	22	144
	Row Pct	6.25	7.64	13.19	34.72	15.28	7.64	15.28	
	Col Pct	37.50	64.71	57.58	62.50	46.81	50.00	44.00	
1	Frequency	15	6	14	30	25	11	28	129
	Row Pct	11.63	4.65	10.85	23.26	19.38	8.53	21.71	
	Col Pct	62.50	35.29	42.42	37.50	53.19	50.00	56.00	
Total		24	17	33	80	47	22	50	273

Frequency Missing = 250

Table of OpioidsPost by RminusL

OpioidsPost RminusL

OpioidsPost	Frequency / Row Pct / Col Pct	-3	-2	-1	0	1	2	3	Total
0	Frequency	20	14	23	66	39	17	43	222

```
          |   9.01 |   6.31 |  10.36 |  29.73 |  17.57 |
7.66 |  19.37 |
          |  83.33 |  82.35 |  69.70 |  82.50 |  82.98 |
77.27 |  86.00 |
---------+--------+--------+--------+--------+--------+-------
-+--------+
     1 |      4 |   3 |  10 |     14 |       8 |       5 |
7 |  51
          |   7.84 |   5.88 |  19.61 |  27.45 |  15.69 |
9.80 |  13.73 |
          |  16.67 |  17.65 |  30.30 |  17.50 |  17.02 |
22.73 |  14.00 |
---------+--------+--------+--------+--------+--------+-------
-+--------+
Total          24         17        33    80         47        22
     50   273

Frequency Missing = 250


Table of BarbituratesPost by RminusL

BarbituratesPost    RminusL

Frequency|
Row Pct  |
Col Pct  |      -3|      -2|       -1|       0|        1|
     2|       3|  Total
---------+--------+--------+--------+--------+--------+-------
-+--------+
     0 |     19 |   15 |      24 |     64 |  33 |      14 |
36 | 205
          |   9.27 |   7.32 |  11.71 |  31.22 |  16.10 |
6.83 |  17.56 |
          |  79.17 |  88.24 |  72.73 |  80.00 |  70.21 |
63.64 |  72.00 |
---------+--------+--------+--------+--------+--------+-------
-+--------+
     1 |      5 |   2 |       9 |     16 |  14 |      8 |
14 |      68
          |   7.35 |   2.94 |  13.24 |  23.53 |  20.59 |
11.76 |  20.59 |
          |  20.83 |  11.76 |  27.27 |  20.00 |  29.79 |
36.36 |  28.00 |
```

```
---------+--------+--------+--------+--------+--------+-------
-+--------+
Total           24       17         33         80        47    22
     50      273

Frequency Missing = 250


Table of TriptansPost by RminusL

TriptansPost   RminusL

Frequency|
Row Pct  |
Col Pct  |      -3|      -2|        -1|       0|        1|
     2|       3|  Total
---------+--------+--------+--------+--------+--------+-------
-+--------+
     0 |      4 |   5 |  13 |     23 |  14 |      9 |     10 |
     78
         |   5.13 |   6.41 |  16.67 |  29.49 |  17.95 |
11.54 |  12.82 |
         |  16.67 |  29.41 |  39.39 |  28.75 |  29.79 |
40.91 |  20.00 |
---------+--------+--------+--------+--------+--------+-------
-+--------+
     1 |     20 |   12 |      20 |     57 |  33 |      13 |
40 | 195
         |  10.26 |   6.15 |  10.26 |  29.23 |  16.92 |
6.67 |  20.51 |
         |  83.33 |  70.59 |  60.61 |  71.25 |  70.21 |
59.09 |  80.00 |
---------+--------+--------+--------+--------+--------+-------
-+--------+
Total          24         17        33    80        47       22
     50   273

Frequency Missing = 250


Table of race by RminusL

race(Race)     RminusL
```

```
Frequency       |
Row Pct         |
Col Pct         |      -3|     -2|       -1|       0|        1|
     2|       3|  Total
-----------------+--------+--------+--------+--------+--------
+--------+--------+
American Indian  |       0 |       1 |   0 |       0 |       0 |
     0 |       0 |       1
or Alaska Native |   0.00 | 100.00 |   0.00 |   0.00 |   0.00
|   0.00 |   0.00 |
                |   0.00 |   7.14 |   0.00 |   0.00 |   0.00 |
0.00 |   0.00 |
-----------------+--------+--------+--------+--------+--------
+--------+--------+
Asian           |    0 |       0 |   1 |       2 |       0 |   0
|    0 |       3
                |   0.00 |   0.00 |  33.33 |  66.67 |   0.00 |
0.00 |   0.00 |
                |   0.00 |   0.00 |   3.70 |   2.86 |   0.00 |
0.00 |   0.00 |
-----------------+--------+--------+--------+--------+--------
+--------+--------+
Black or African |       1 |       2 |       0 |   2 |       2
|      1 |     2 |     10
/American       |  10.00 |  20.00 |   0.00 |  20.00 |  20.00 |
10.00 |  20.00 |
                |   5.00 |  14.29 |   0.00 |   2.86 |   4.44 |
4.76 |   4.35 |
-----------------+--------+--------+--------+--------+--------
+--------+--------+
White           |    19 |      11 |  26 |      66 |      43 |  20
|    44 |    229
                |   8.30 |   4.80 |  11.35 |  28.82 |  18.78 |
8.73 |  19.21 |
                |  95.00 |  78.57 |  96.30 |  94.29 |  95.56 |
95.24 |  95.65 |
-----------------+--------+--------+--------+--------+--------
+--------+--------+
Other           |    0 |       0 |   0 |       0 |       0 |   0
|    0 |       0
                |    . |       . |   . |       . |       . |   .
|      . |
                |   0.00 |   0.00 |   0.00 |   0.00 |   0.00 |
0.00 |   0.00 |
```

```
----------------+-------+-------+-------+-------+--------
+--------+--------+
Total              20        14        27    70        45
21        46   243

Frequency Missing = 280


Table of marital_status by RminusL

marital_status(Marital Status)     RminusL

Frequency|
Row Pct  |
Col Pct  |      -3|      -2|       -1|       0|       1|
     2|       3|  Total
---------+--------+--------+--------+--------+--------+-------
-+--------+
Married  |     17 |      10 |      19 |     46 |  29 |      13
|     31 |     165
          |  10.30 |   6.06 |  11.52 |  27.88 |  17.58 |
7.88 |  18.79 |
          |  70.83 |  62.50 |  63.33 |  58.23 |  61.70 |
61.90 |  63.27 |
---------+--------+--------+--------+--------+--------+-------
-+--------+
Single   |      5 |      4 |       6 |     30 |   16 |      7
|     11 |     79
          |   6.33 |   5.06 |   7.59 |  37.97 |  20.25 |
8.86 |  13.92 |
          |  20.83 |  25.00 |  20.00 |  37.97 |  34.04 |
33.33 |  22.45 |
---------+--------+--------+--------+--------+--------+-------
-+--------+
Divorced |     2 |      1 |   5 |       3 |      2 |   1 |
     7 |     21
          |   9.52 |   4.76 |  23.81 |  14.29 |   9.52 |
4.76 |  33.33 |
          |   8.33 |   6.25 |  16.67 |   3.80 |   4.26 |
4.76 |  14.29 |
---------+--------+--------+--------+--------+--------+-------
-+--------+
Widowed  |      0 |      0 |       0 |      0 |   0 |       0
|      0 |     0
```

```
         |      . |      . |       . |      . |   . |       .
|      . |
          |   0.00 |   0.00 |   0.00 |   0.00 |   0.00 |
0.00 |   0.00 |
---------+--------+--------+--------+--------+--------+-------
-+--------+
Other     |      0 |      1 |       0 |      0 |   0 |       0
|      0 |      1
          |   0.00 | 100.00 |   0.00 |   0.00 |   0.00 |
0.00 |   0.00 |
          |   0.00 |   6.25 |   0.00 |   0.00 |   0.00 |
0.00 |   0.00 |
---------+--------+--------+--------+--------+--------+-------
-+--------+
Total          24         16        30    79         47       21
     49   266

Frequency Missing = 257


Table of education by RminusL

education(Education)     RminusL

Frequency       |
Row Pct         |
Col Pct         |      -3|     -2|       -1|       0|        1|
     2|       3|  Total
-----------------+--------+--------+--------+--------+--------
+--------+--------+
Some high school |       0 |       0 |      0 |   3 |       0
|      0 |     0 |       3
 or less         |   0.00 |   0.00 |   0.00 | 100.00 |   0.00
|   0.00 |   0.00 |
                |   0.00 |   0.00 |   0.00 |   4.48 |   0.00 |
0.00 |   0.00 |
-----------------+--------+--------+--------+--------+--------
+--------+--------+
High school grad |       4 |       5 |      8 |   5 |      7 |
     2 |       7 |     38
uate or GED     |  10.53 |  13.16 |  21.05 |  13.16 |  18.42 |
5.26 |  18.42 |
                |  22.22 |  41.67 |  28.57 |   7.46 |  16.28 |
11.11 |  14.58 |
```

```
----------------+--------+--------+--------+--------+--------
+--------+--------+
Some college   |      4 |      2 |       6 |      19 |   7 |
     4 |     11 |   53
               |   7.55 |   3.77 |  11.32 |  35.85 |  13.21 |
7.55 |  20.75 |
               |  22.22 |  16.67 |  21.43 |  28.36 |  16.28 |
22.22 |  22.92 |
----------------+--------+--------+--------+--------+--------
+--------+--------+
Graduate or coll |  10 |      5 |     14 |   40 |      29 |
12 |      30 | 140
ege or postgradu |   7.14 |   3.57 |  10.00 |  28.57 |  20.71
|   8.57 |  21.43 |
ate degree     |  55.56 |  41.67 |  50.00 |  59.70 |  67.44 |
66.67 |  62.50 |
----------------+--------+--------+--------+--------+--------
+--------+--------+
Total              18        12        28    67        43
18        48   234

Frequency Missing = 289


Table of employment by RminusL

employment(Employment status)      RminusL

Frequency  |
Row Pct   |
Col Pct   |      -3|     -2|       -1|       0|        1|
     2|       3|  Total
-----------+--------+--------+--------+--------+--------+-----
---+--------+
Employed   |     17 |    5 |  14 |     44 |  28 |      12 |
30 | 150
          |  11.33 |   3.33 |   9.33 |  29.33 |  18.67 |
8.00 |  20.00 |
          |  85.00 |  45.45 |  51.85 |  64.71 |  66.67 |
75.00 |  66.67 |
-----------+--------+--------+--------+--------+--------+-----
---+--------+
Unemployed |   0 |      1 |   4 |       6 |      3 |   1 |
     3 |     18
```

```
          |   0.00 |   5.56 |  22.22 |  33.33 |  16.67 |
5.56 |  16.67 |
          |   0.00 |   9.09 |  14.81 |   8.82 |   7.14 |
6.25 |   6.67 |
-----------+--------+--------+--------+--------+--------+-----
---+--------+
Disabled   |      1 |    1 |        4 |      3 |   2 |       1
|      2 |     14
           |   7.14 |   7.14 |  28.57 |  21.43 |  14.29 |
7.14 |  14.29 |
          |   5.00 |   9.09 |  14.81 |   4.41 |   4.76 |
6.25 |   4.44 |
-----------+--------+--------+--------+--------+--------+-----
---+--------+
Student   |      0 |      1 |        1 |      5 |   3 |       1
|      4 |     15
          |   0.00 |   6.67 |   6.67 |  33.33 |  20.00 |
6.67 |  26.67 |
          |   0.00 |   9.09 |   3.70 |   7.35 |   7.14 |
6.25 |   8.89 |
-----------+--------+--------+--------+--------+--------+-----
---+--------+
Homemaker  |      2 |    1 |        2 |      7 |   3 |       1
|      4 |     20
          |  10.00 |   5.00 |  10.00 |  35.00 |  15.00 |
5.00 |  20.00 |
          |  10.00 |   9.09 |   7.41 |  10.29 |   7.14 |
6.25 |   8.89 |
-----------+--------+--------+--------+--------+--------+-----
---+--------+
Retired   |      0 |      2 |        2 |      3 |   3 |       0
|      2 |     12
          |   0.00 |  16.67 |  16.67 |  25.00 |  25.00 |
0.00 |  16.67 |
          |   0.00 |  18.18 |   7.41 |   4.41 |   7.14 |
0.00 |   4.44 |
-----------+--------+--------+--------+--------+--------+-----
---+--------+
Total          20         11        27    68         42       16
     45   229

Frequency Missing = 294
```

Table of headache_dx by RminusL

headache_dx(Principle Headache Diagnosis)   RminusL

Frequency / Row Pct / Col Pct	-3	-2	-1	0	1	2	3	Total
Migraine	24	17	33	80	47	22	50	273
	8.79	6.23	12.09	29.30	17.22	8.06	18.32	
	100.00	100.00	100.00	100.00	100.00	100.00	100.00	
Total	24	17	33	80	47	22	50	273

Frequency Missing = 250

Table of hand by RminusL

hand(Handedness)   RminusL

Frequency / Row Pct / Col Pct	-3	-2	-1	0	1	2	3	Total
Left	7	2	0	9	2	0	5	25
	28.00	8.00	0.00	36.00	8.00	0.00	20.00	
	30.43	13.33	0.00	12.00	4.88	0.00	10.87	
Right	16	13	27	65	39	20	41	221

```
               |   7.24 |   5.88 |  12.22 |  29.41 |  17.65 |
9.05 |  18.55 |
               |  69.57 |  86.67 | 100.00 |  86.67 |  95.12 |
100.00 |  89.13 |
-----------------+--------+--------+--------+--------+--------
+--------+--------+
Right preference |       0 |       0 |      0 |   0 |       0
|      0 |     0 |       0
               |    . |      . |   . |      . |   . |       .
|      . |
               |   0.00 |   0.00 |   0.00 |   0.00 |   0.00 |
0.00 |   0.00 |
-----------------+--------+--------+--------+--------+--------
+--------+--------+
Ambidextrous   |      0 |      0 |   0 |      1 |       0 |
0 |       0 |       1
               |   0.00 |   0.00 |   0.00 | 100.00 |   0.00 |
0.00 |   0.00 |
               |   0.00 |   0.00 |   0.00 |   1.33 |   0.00 |
0.00 |   0.00 |
-----------------+--------+--------+--------+--------+--------
+--------+--------+
Total               23         15        27    75         41
20         46   247

Frequency Missing = 276

~~~~~~~~~~~~~~~~~~~~~~~~~~~~~~~~~~~~~~~~~~~~~~~~~~~~~~~~~~~~~~
~~~~~~~~~~~~~~~~~~~~~~~~~~~~~~~~~~~~~~~~~~~~~~~~~~~~~~~~~~~~

 Jonckheere-Terpstra Test for trend p-values

Table sex * RminusL                0.3297
Table White * RminusL              0.3275
Table ethnicity * RminusL          0.5453
Table Married * RminusL            0.7814
Table CollegeGrad * RminusL        0.1174
Table Employed * RminusL           0.6424
Table aura * RminusL               0.5733
Table RightHanded * RminusL        0.0736
Table Substance * RminusL          0.6812
Table Anxiety * RminusL            0.3265
Table Depression * RminusL         0.5142
Table Bipolar * RminusL            0.7507
```

```
Table Eating * RminusL             0.5579
Table Psychotic * RminusL          0.0863
Table PTSD * RminusL               0.0048
Table OpiodsPrior * RminusL        0.2507
Table BarbituratesPrior * RminusL  0.3609
Table TriptansPrior * RminusL      0.2693
Table OpioidsPost * RminusL        0.3893
Table BarbituratesPost * RminusL   0.1298
Table TriptansPost * RminusL       0.7369
Correlation of education level and RminusL is 0.11868, p=
0.0700, n=234

~~~~~~~~~~~~~~~~~~~~~~~~~~~~~~~~~~~~~~~~~~~~~~~~~~~~~~~~~~~~~~
~~~~~~~~~~~~~~~~~~~~~~~~~~~~~~~~~~~~~~~~~~~~~~~~~~~~~~~~~~~~

This helps to see the direction of the trend:

                                        Analysis Variable :
RminusL


     PTSD    Obs         Mean      Std Dev   N          Median
     Minimum        Maximum
--------------------------------------------------------------
--------------------------------------------
          0    494       0.4513619      1.7607283 257
     0    -3.0000000     3.0000000
          1     29       -0.8750000     1.7078251      16
-0.5000000     -3.0000000     3.0000000
--------------------------------------------------------------
--------------------------------------------
```

PTSD sufferers differ from nonPTSD individuals whereby sufferers are on the left side on average, and non-sufferers are slightly on the right. Same can be seen in the crosstabulation above (p<0.005). Note that the N with PTSD is only 16.
